# New insights into temperature-impacted mycovirus-fungus interactions regulated by a microRNA in *Lentinula edodes*

**DOI:** 10.1128/jvi.00084-25

**Published:** 2025-08-20

**Authors:** Chun-Xi Liu, Meng-Pei Guo, Jun-Zhuo Zhou, Yi-Jia Sun, Yin-Bing Bian, Zhang-Yi Xu

**Affiliations:** 1 Institute of Applied Mycology, Huazhong Agricultural University 47895, Wuhan, China; 2 Key Laboratory of Agro-Microbial Resource and Development (Ministry of Agriculture), Huazhong Agricultural University 47895, Wuhan, China; Iowa State University, Ames, Iowa, USA

**Keywords:** *Lentinula edodes*, *Lentinula edodes* mycovirus HKB, heat stress, microRNA, interaction

## Abstract

The onset of symptoms in virus-infected mushrooms merits investigation. In this study, the influence of heat stress (HS) on the interaction between *Lentinula edodes* mycovirus HKB (LeV) and its mushroom host, *Lentinula edodes*, was explored, revealing that HS treatment facilitated LeV replication and compromised the host’s thermotolerance at full growth of mycelia. Transcriptomic analysis showed that fewer genes responded to HS, and thermotolerance-related genes were downregulated when the virus infection occurred. A host microRNA, led-milR-21, proven as a negative regulator of the growth rate, thermotolerance, and resistance against *Trichoderma atroviride* of *L. edodes* mycelia by functional analyses, could be induced by both LeV infection and HS. Tobacco transient expression experiments with qRT-PCR confirmation demonstrated that led-milR-21 targeted a thermotolerance-related transcription factor *LE01Gene01783*. Further analysis showed that genes associated with tryptophan metabolism, arginine and proline metabolism, valine/leucine and isoleucine degradation, protein processing in the endoplasmic reticulum, fatty acid degradation, and glycerolipid metabolism may play roles in the responses of *L. edodes* to LeV infection and HS. Based on these findings, a putative mechanism was proposed to elucidate the interplay among temperature, LeV, and *L. edodes*. This study deepens our understanding of how fruit body-forming fungi respond to viral infection and abiotic stress, providing insights into potential virus-host interactions in a scenario of global warming.

**IMPORTANCE:**

This is the first report of a fungal miRNA being induced by a mycovirus. In *Lentinula edodes*, upon LeV infection under heat stress, LeV replication surges, triggering degradation of the LeV genome by *DCL1*, particularly on its 5′-UTR end and ORF1 upstream regions. *DCL1* and several other RNAi key genes (such as *LeAGO8*, *LeRDR1*, *LeRDR5*, and *LeRDR6*) are also possibly recruited during the thermotolerance-related host microRNA (*led-milR-21*) formation, leading to its increased production. Consequently, led-milR-21-dependent silencing of *LE01Gene01783* occurs upon LeV infection, diminishing the heat repair capacity. This study deepens our understanding of how fruiting body-forming fungi respond to viral infection and abiotic stress, providing insights into potential virus-host interactions in a scenario of global warming.

## INTRODUCTION

Global warming and climate volatility pose growing challenges to crop cultivation and disease management. Mushroom-forming fungi are complex multicellular organisms that produce large, economically significant fruiting bodies. The yield and quality of fruiting bodies are highly sensitive to temperature fluctuations. Optimal temperatures are critical for mycelial growth and nutrient accumulation during the fungal vegetative stage, which are prerequisites for healthy fruit development. Previous studies have shown that heat stress (HS) severely inhibits mycelial growth, damages cellular structures and metabolism, increases susceptibility to microbial pathogens (e.g., *Trichoderma asperellum*, *T. harzianum*, *T. atroviride*), and may even trigger apoptotic-like cell death (1, 2). HS also impairs primordial development and dramatically reduces production (3). To counteract HS, mushrooms have evolved sophisticated molecular mechanisms, including antioxidant defense systems to neutralize excessive reactive oxygen species (ROS) (4); trehalose accumulation to stabilize enzymes, proteins, and cellular structures (5, 6); heat shock proteins (HSPs), such as Hsp40, Hsp70, and Hsp90, which repair damaged proteins and enhance antioxidant enzyme activity (7–9); signaling molecules (e.g., Ca²^+^, H₂S, NO) have been found to be involved in HS signal transduction (10, 11).

Mycoviruses are ubiquitous in fungi and typically persist asymptomatically, reflecting a long-term co-evolutionary balance (12). However, certain mycoviruses alter host phenotypes, including hypovirulence, endophytic trait regulation, metabolite production, and drug resistance. Some transform pathogenic fungi into beneficial endophytes that protect plants, while others exacerbate virulence (13–18). Studies on mycovirus diseases typically focus on pathogen-host interactions; however, environmental factors, especially temperature, can also significantly influence infection outcomes (19, 20).

Research aimed at understanding the shift from latent to symptomatic viral infections has largely sought to enhance the effectiveness of mycoviruses as biocontrol agents against phytopathogenic fungi (18). However, such a transition can be economically disastrous to cultivated mushrooms by imposing great quality reduction and yield losses (21). Although the first mycovirus was identified in *Agaricus bisporus* (22), its definitive link to mushroom diseases like La France disease or X-disease has not been conclusively determined (14, 21). Subsequent reports of viral infections in other mushroom species like *Pleurotus* spp., *Lentinula edodes*, and *Flammulina velutipes* have not elucidated the impact of the viruses on their fungal hosts either (23–28). In addition, the role of environmental factors played in the process of mycovirus infection in mushrooms has yet to be studied.

*L. edodes* is the most widely cultivated edible fungus in the world and has various antiviral and immunomodulating properties (29, 30). Numerous mycoviruses have been identified in symptomatic or asymptomatic samples of *L. edodes* (23, 27, 28, 31–34). Among these, Lentinula edodes mycovirus HKB (LeV), isolated from an asymptomatic Japanese strain, is notable as the first mycovirus with a fully sequenced genome (23). LeV, proposed to belong to the family “Phlegivirus,” possesses a monopartite dsRNA genome (~11 kbp) encoding two overlapping open reading frames (ORFs): ORF1 (NUDIX-hydrolase motif) and ORF2 (RdRp motif) (35). Here, we demonstrate for the first time that HS shifts LeV infection in *L. edodes* from cryptic to symptomatic. We further elucidate temperature-dependent virus-host interactions mediated by miRNA regulation. This work advances our understanding of fungal responses to viral infections and abiotic stress, offering critical insights for predicting virus-host dynamics under global warming.

## RESULTS

### Influences of heat stress on LeV-*L. edodes* interactions

**Fig 1:**
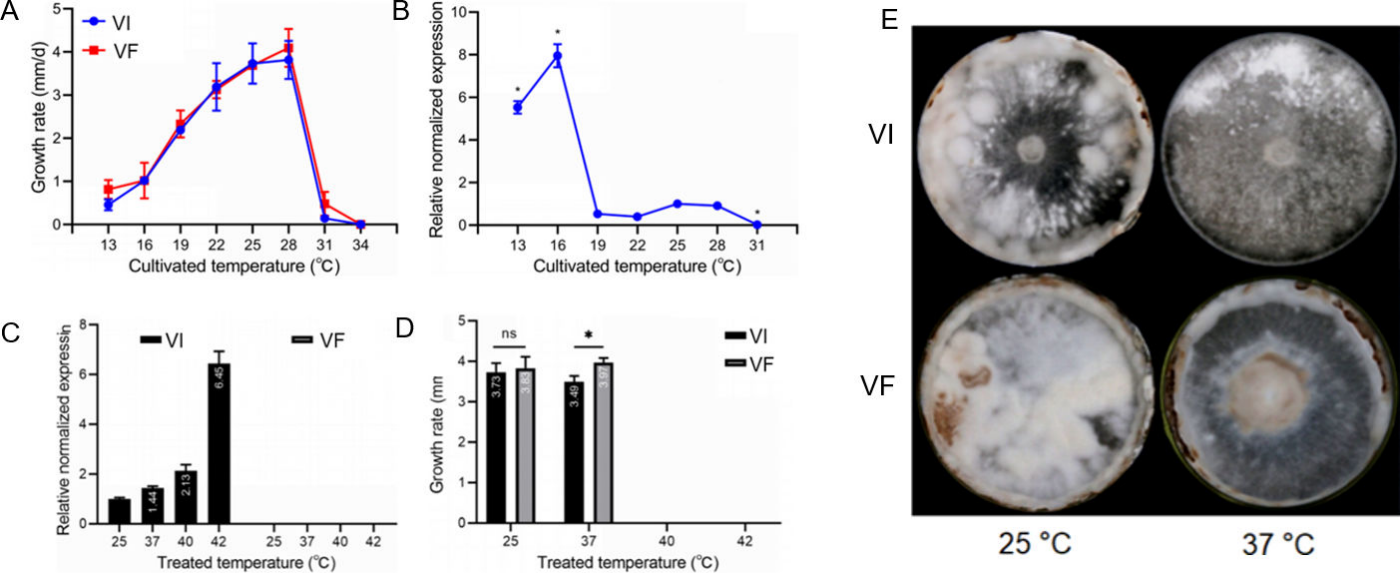
The effect of LeV on the growth of *L. edodes* and the relative expression levels of the *RdRp* gene under different temperatures. (A) Effect of LeV on the mycelial growth of *L. edodes* when the mycelia were long cultured at the same temperature. (B) Relative expression levels of the *RdRp* gene in mycelia that were long cultured at the same temperature. (C) Relative expression levels of the *RdRp* gene in mycelia that grew fully on the plate at 25°C and were then treated with different HS for 24 h. (D) The growth rate of regeneration cultured under different temperature treatments. (E) Colony phenotype being re-cultured at 25°C for 5 d in darkness and then a regimen of alternating 12 hour periods of light exposure at 300 Lux and darkness for 10 d after HS treatment (37°C for 24 h). ns represents no significant difference from that of 25°C; * represents significant difference, *P* < 0.05.

To test the effects of temperature on the VI (LeV-infected strain), using VF (LeV-free strain) as a control, the VI strain was cultured at 19°C, 22°C, 25°C, 28°C, 31°C, and 34°C. Subsequently, the mycelial growth rates and LeV *RdRp* mRNA expressions were measured by RT-qPCR. The results showed that between 13°C and 28°C, the growth rates of strains VI and VF increased, then sharply declined at 31°C and ceased completely at 34°C, with no significant difference between strains (Fig. 1A). RT-qPCR analysis revealed higher LeV RdRp mRNA expression levels at lower temperatures (13°C and 16°C), which were inhibited at 31°C (Fig. 1B). However, after full mycelial growth at 25°C and subsequent HS treatments, LeV RdRp mRNA expression increased (Fig. 1C). The HS-induced regeneration growth rate of VI was significantly lower than that of VF (Fig. 1D). Colonies of the two strains showed distinct browning and hyphal aggregation after a 24 hour treatment at 37°C followed by a 26-day culture at 25°C (Fig. 1E). The mycelia of both strains did not grow at 40°C or 42°C (Fig. 1D).

**Fig 2:**
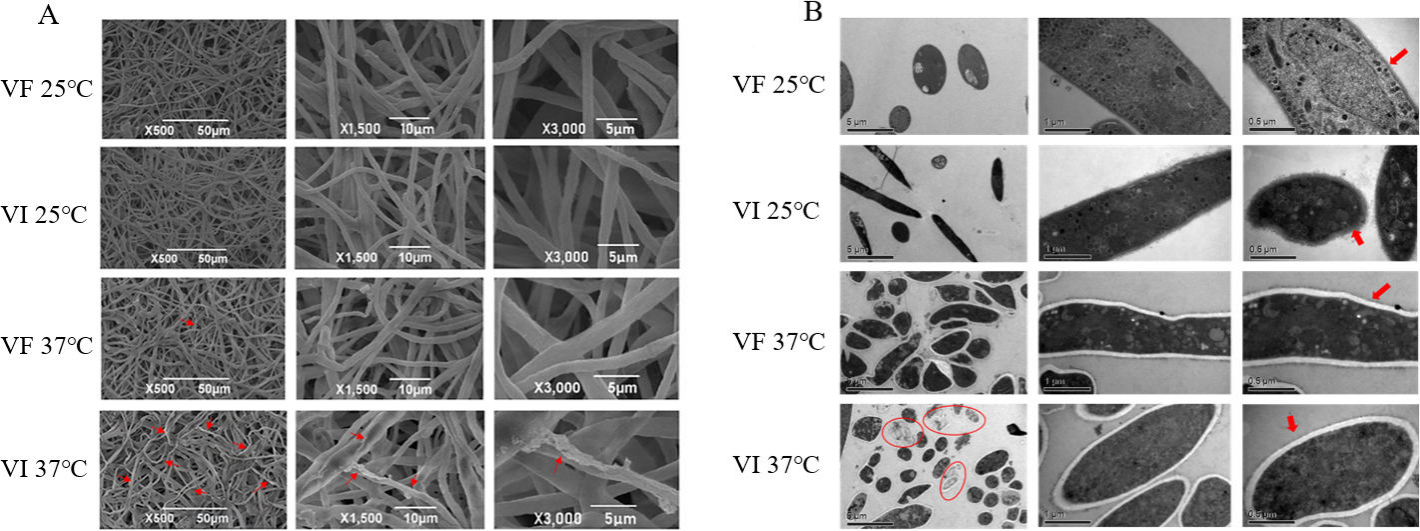
Electron microscopic observation results of VF and VI treated by 25°C treatment (control, the mycelia cultured at 25°C) and HS treatment (the mycelia grew fully on the plate at 25°C and then treated by 37°C for 48 h). (A) Scanning electron microscope observations and (B) transmission electron microscope observations.

SEM results showed distinct mycelial responses between VI and VF: the VF strain maintained a uniform mycelial shape with minimal shrinkage, while the VI strain exhibited shrinkage, twisting, and cell membrane damage accompanied by cell-content leakage (Fig. 2A). TEM revealed clear organelle membranes and intact cell structures for both strains at 25°C. By contrast, after HS treatment, both strains exhibited significantly thickened cell walls. The cell contents of VF became turbid, though their organelle membranes remained clear, whereas VI showed increased turbidity, indistinct organelle membranes, and some cells ruptured, leading to cell-content leakage (Fig. 2B).

### Co-influence of LeV and HS on thermotolerance-related gene expression in *L. edodes*

To investigate the differences in gene expression response between VI and VF after HS, RNA-seq data from six samples (as described in Materials and Methods) were further obtained (GenBank no. PRJNA1120119) and analyzed. Sequencing statistics are detailed in Table S1, as well as the principal component analysis (PCA), and the differentially expressed genes (DEGs) are presented in Fig. S1 and S2, respectively. At the T1 stage, VI showed a significantly reduced number of DEGs compared to VF (1,056 vs. 2,030), but this trend reversed at the T2 stage (Table S2). Activities of HSPs and transcription factors (TFs), which are potentially related to thermotolerance, were higher in VF than in VI at the T1 stage, with no significant difference observed at the T2 stage (Table S3; Fig. S3 and S4). The DEGs of VI were enriched in only three metabolic pathways at the T1 stage: ribosome biogenesis, glycolysis and gluconeogenesis, and chaperones/folding catalysts. By contrast, VF showed enrichment in 10 pathways, including homologous recombination, amino acid-related enzymes, tryptophan metabolism, DNA repair and recombination proteins, cysteine and methionine metabolism, DNA replication, amino acid metabolism, basic transcription factors, and replication/repair (Fig. S5A and B). At the T2 stage, VF was enriched in amino acid metabolism, lipid metabolism, and carbohydrate metabolism, whereas VI exhibited enrichment in energy metabolism, chaperones/folding catalysts, peptides, oxidative phosphorylation, and glycolysis and gluconeogenesis (Fig. S5C and D). These results suggest that LeV may impair VI’s thermal repair capacity post-HS, leading to decreased mycelial growth rates at the T1 stage and indirectly affecting mycelial aggregation and browning at the T2 stage.

Weighted gene co-expression network analysis (WGCNA) clusters co-expressed genes into color-coded modules and identifies their statistical associations with experimental traits, thereby uncovering biologically relevant gene networks (36). Using WGCNA, all samples with different expression patterns of genes were clustered into 12 co-expression modules, each assigned a unique color, and we identified key genes in *L. edodes* responsive to both HS and LeV infection, which clustered into five distinct color-coded modules based on their expression patterns, with the MEsienna3 module having a high Pearson correlation coefficient (0.92, *P* = 3e-09) (Fig. S6A). The MEsienna3 module contains 1,039 genes, 370 of which were DEGs in VI or VF strains (281 in VI, 89 in VF). GO and KEGG analyses performed on these 1039 genes highlighted enrichment in molecular chaperones and folding catalysts (including several *HSPs*), protein processing in the endoplasmic reticulum, and genes related to cell membrane structure, glycerophospholipid metabolism, indicating that HS primarily damaged the cell membranes of VI and VF, with a greater impact on VI than VF (Fig. S6B).

### Characteristics of *L. edodes* immune response to LeV post-HS

**Fig 3:**
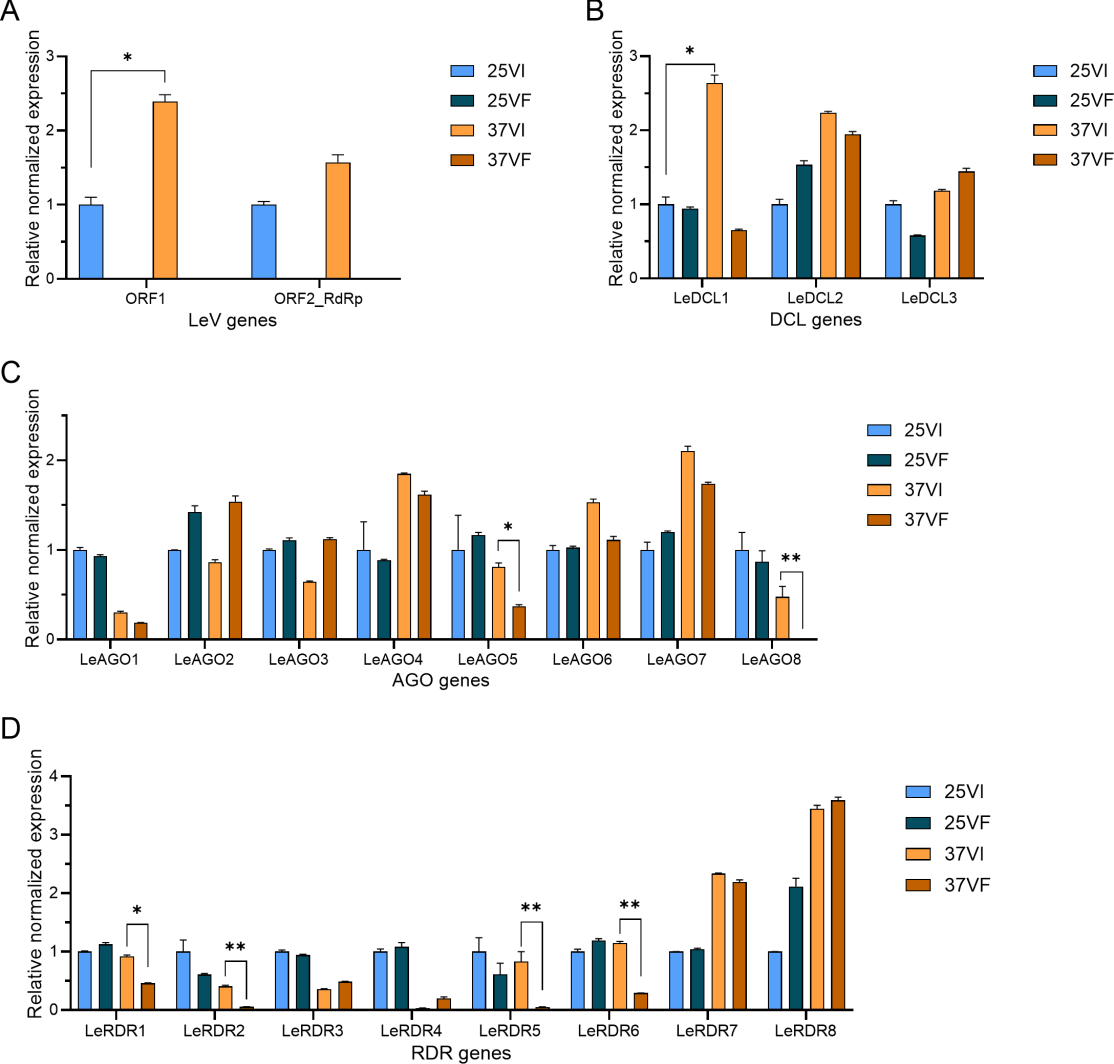
Relative expression levels of the genes encoding two ORFs of LeV and RNAi key genes in *L. edodes*. (A) Relative expression levels of genes encoding two ORFs of LeV. (B) Relative expression levels of *DCL*. (C) Relative expression levels of *AGO*. (D) Relative expression levels of *RDR*. * represents significant differences, *P* < 0.05; ** represents extremely significant differences, *P* < 0.01.

**Fig 4:**
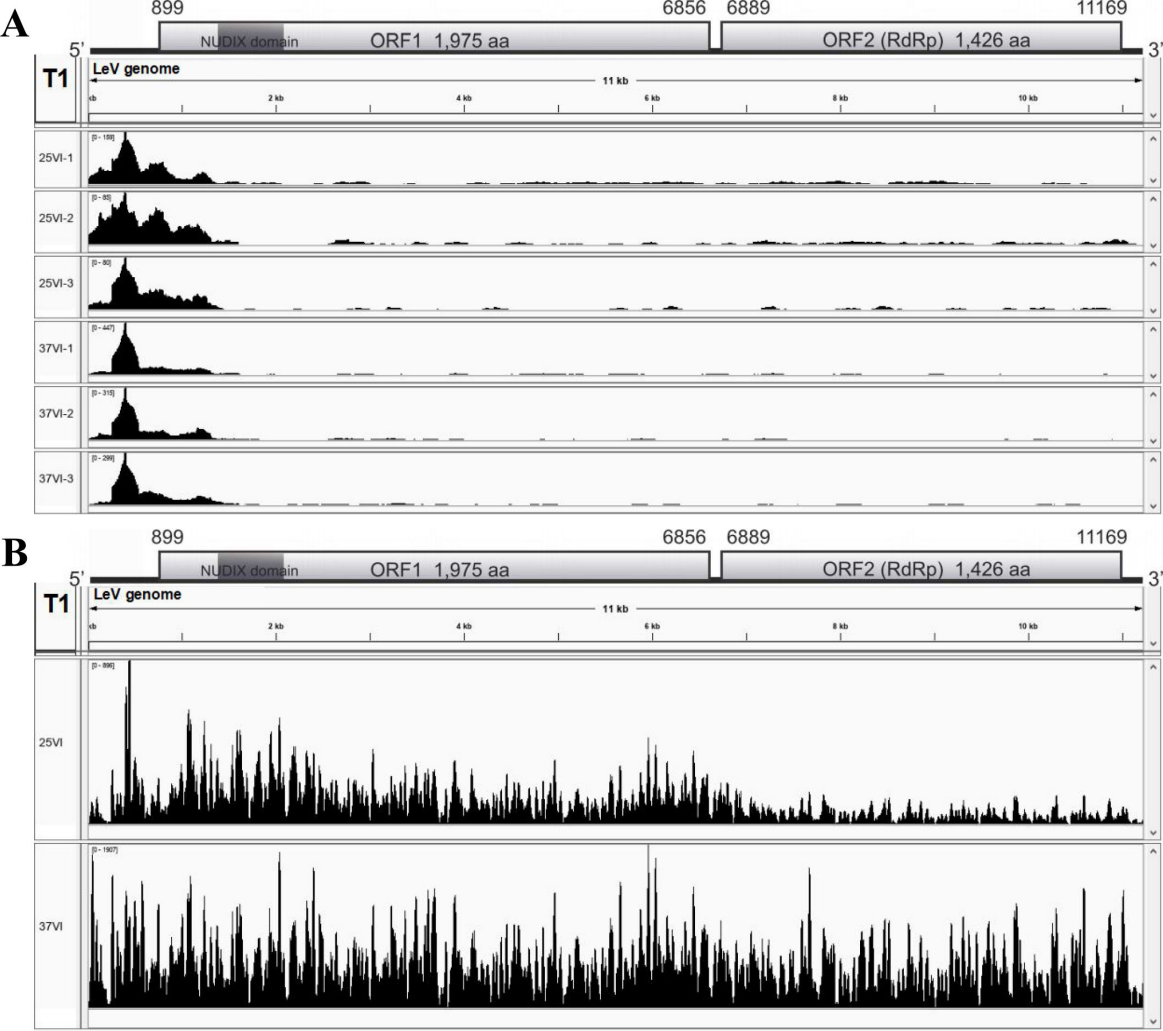
The mapping profiles of the sequenced data to the LeV genome. (A) The distribution profile of LeV-derived mRNA reads at the T1 stage. (B) The distribution profile of the sequenced virus-derived small interfering RNAs (vsiRNAs) at the T1 stage.

After HS, the expression of two ORFs encoding LeV genes increased in the VI, with viral-derived mRNA reads predominantly located upstream of ORF1 in the LeV genome (GenBank accession number AB429556) (Fig. 3A and 4A). The quantity of virus-derived small interfering RNA (vsiRNA) surged from 896 to 1,907 reads. In VI treated at 25°C (25VI), vsiRNA clusters were concentrated in the center of the 5′ untranslated region (5′-UTR) and around ORF1, contrasting with the uniform distribution observed in VI treated at 37°C (37VI), particularly showing increased vsiRNA abundance near ORF2-RdRp (Fig. 4B). A negative correlation between mRNA read abundance and vsiRNA levels at identical genomic positions was also detected. These findings suggest that HS may enhance LeV replication and alter the degradation mechanism of LeV mRNA molecules.

In all, 168 vsiRNAs that increased by over 10-fold after HS were identified. Using the psRobot software, we predicted their potential binding sites based on *L. edodes* cDNA sequences. This analysis revealed 83 vsiRNA-target mRNA pairs, involving 52 unique vsiRNAs and 83 target mRNAs. Notably, among these target mRNAs, only two genes (*LE01Gene14484* and *LE01Gene14484*) were significantly downregulated post-HS, with transcript per million (TPM) values ranging from 1 to 6 (Fig. 4C). These results suggest that vsiRNAs may have a limited direct impact on *L. edodes* gene expression under the tested conditions. After HS, we analyzed the expression profiles of key RNAi pathway genes in *L. edodes*. Notable changes were observed in one Dicer-like (DCL) gene (*LeDCL1*), two Argonaute (AGO) genes (*LeAGO5* and *LeAGO8*), and four RNA-dependent RNA polymerase (RDR) genes (*LeRDR1*, *LeRDR2*, *LeRDR5*, and *LeRDR6*) (Fig. 3B through D). In addition, an integrated analysis of sRNA sequencing data and stem-loop RT-qPCR validation predicted a microRNA-like RNA (milRNA, designated led-milR-21) potentially associated with both HS and LeV infection (Fig. S7). The precursor sequence of led-milR-21 (designated Pre-milR21) is a 502 bp non-coding region, and its predicted secondary structure is illustrated in Fig. S8. led-milR-21 was the most abundant milRNA in the sRNA sequencing data set. Post-HS, RT-qPCR and counts per million (CPM) analysis revealed that led-milR-21 expression showed no significant change in the VI but was significantly downregulated in the VF (approximately fourfold decrease, *P<0.05). These results suggest that LeV may exert a positive regulatory effect on led-milR-21 levels, counteracting the HS-induced downregulation observed in VI.

### Verification of the correlation among LeV, *led-milR-21*, and *DCL1* influenced by HS

**Fig 5:**
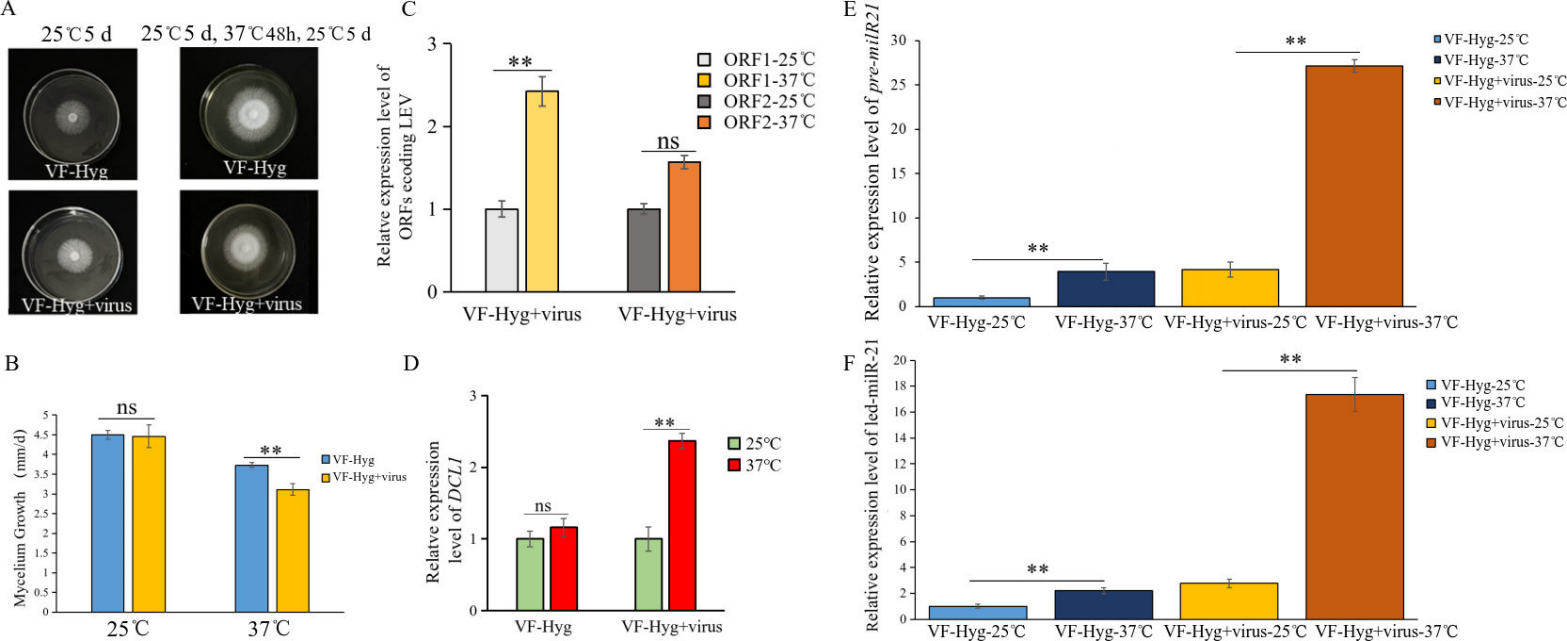
Verification of the effect of LeV on *L. edodes* after HS and the correlation among LeV, *led-milR-21*, and *DCL1* influenced by HS. (A) The colony phenotypes of VF-Hyg (reconstructed from a hygromycin-resistant LeV-free strain) and VF-Hyg +virus (reconstructed from a hygromycin-resistant LeV-infected strain). (B) The mycelial growth rates of VF-Hyg and VF-Hyg + virus under different temperature treatments. (C) Relative expression levels of genes encoding two ORFs of VF-Hyg + virus under different temperature treatments. (D) Relative expression levels of *DCL1* of VF-Hyg and VF-Hyg + virus under different temperature treatments. (E) Relative expression levels of *Pre-milR21*. (F) Relative expression levels of Led-milR-21 of VF-Hyg and VF-Hyg + virus under different temperature treatments.

To validate the aforementioned findings, a hygromycin-resistant LeV-free strain (VF-Hyg) was generated by introducing the hygromycin resistance gene (Hyg) into the VF strain via *Agrobacterium tumefaciens*-mediated transformation. Subsequently, horizontal transmission of LeV from the VI to the VF-Hyg through hyphal contact produced a new hygromycin-resistant LeV-infected strain (VF-Hyg + LeV) (Fig. S9A, C, and D). The introduction of Hyg did not significantly alter the biological characteristics of the VF strain (Fig. S9B). The reconstituted LeV-infected strain (VF-Hyg + LeV) and its virus-free counterpart (VF-Hyg) were used to assess the impact of LeV on *L. edodes* thermotolerance. After 48 hours of treatment at 37°C, the colony diameter (Fig. 5A) and mycelial growth rate (Fig. 5B) of VF-Hyg +LeV were significantly smaller and lower than those of VF-Hyg, confirming that LeV compromises fungal thermotolerance. Post-HS, the expression of two LeV ORFs (Fig. 5C) and *LeDCL1* (Fig. 5D) increased in VF-Hyg + LeV compared to VF-Hyg. These results validate HS-induced LeV replication and the dual regulation of *LeDCL1* by both LeV infection and HS. Furthermore, HS treatment upregulated the expression of *Pre-milR21* (Fig. 5E) and mature *led-milR-21* (Fig. 5F) in both VF-Hyg + LeV and VF-Hyg strains compared to the 25°C control. Notably, the LeV-infected strain (VF-Hyg + LeV) exhibited a significantly higher expression of *Pre-milR21* and *led-milR-21* than the virus-free strain (VF-Hyg), suggesting LeV potentiates milRNA accumulation under HS. Collectively, these data demonstrate an HS-modulated interplay among LeV, *Pre-milR21*, *led-milR-21*, and *LeDCL1*.

### Verification of led-milR-21 functions in LeV*-L. edodes* interaction

To investigate the role of *led-milR-21* in the interaction between LeV and *L. edodes* post-HS, overexpression (OEPre) and short tandem target mimic (STTM) vectors targeting the precursor gene (*Pre-milR21*) were constructed and transformed into VI via *Agrobacterium tumefaciens*-mediated transformation. Successful transformants with *Pre-milR21* overexpression (OEPre) or silencing (STTM) were generated and validated (Fig. S10). An empty vector control strain (VI-CK) was established by transforming the VI strain with the backbone plasmid (pCAMBIA1300-g) without an insert. The parental VI strain, VI-CK, and transformants showing the highest (OEPre) or lowest (STTM) led-milR-21 expression levels were subjected to HS treatment (37°C for 48 h) for subsequent analyses.

**Fig 6:**
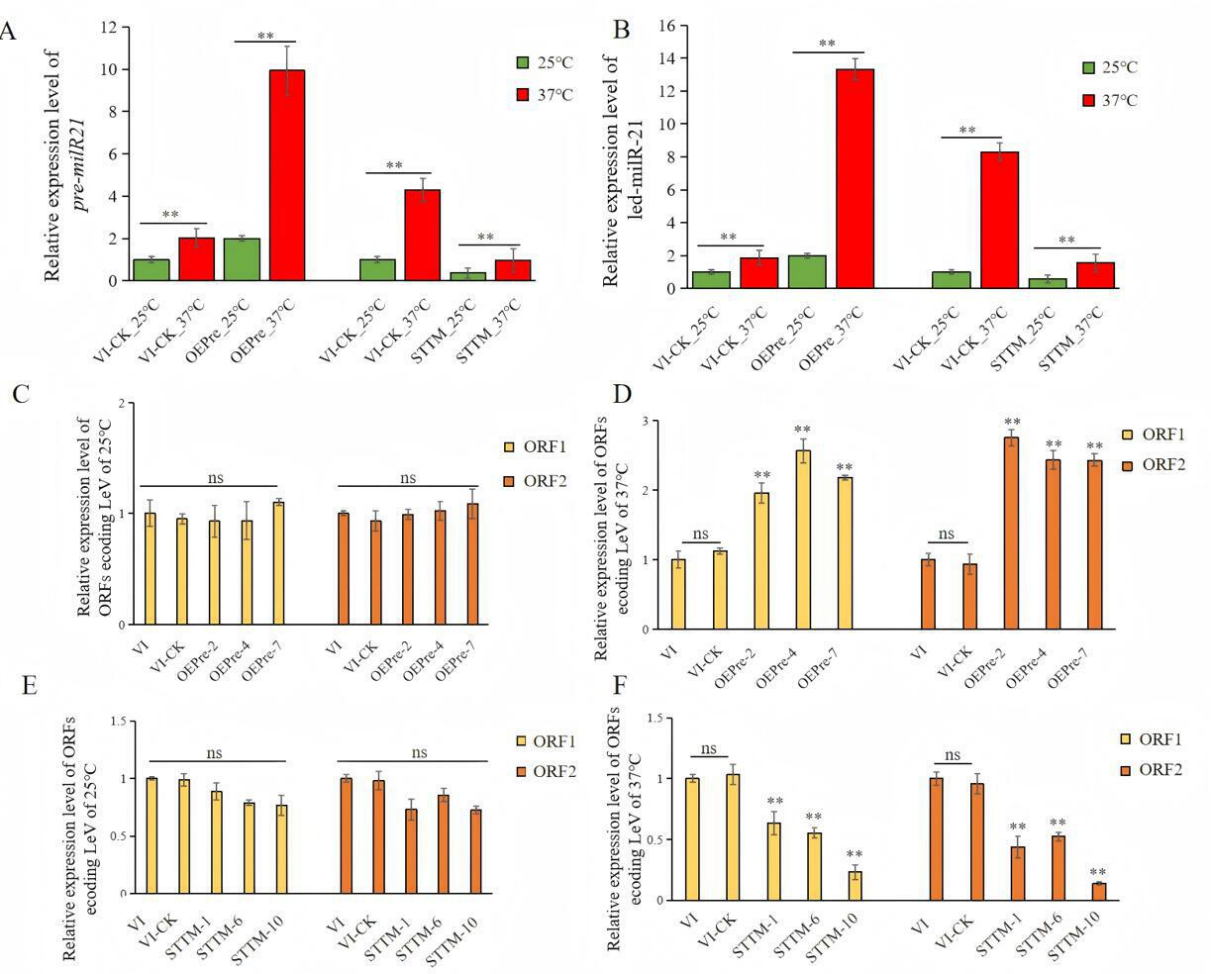
Relative expression levels of genes encoding two ORFs of LeV, *premilR-21*, and *led-milR-21* of the OEPre and STTM transformants under different temperature treatments. (A) RT-qPCR analysis the relative expression levels of *Pre-milR21* in VI-CK, the OEPre and STTM transformants. (B) Stem-loop RT-qPCR analysis of the relative expression levels of *led-milR-21* in VI-CK, the OEPre and STTM transformants. (C and D) RT-qPCR analysis of the relative expression levels of genes encoding two ORFs of LeV in VI-CK and the OEPre transformants treated by 25°C (C) and 37°C (D), respectively. (E and F) RT-qPCR analysis of the relative expression levels of genes encoding two ORFs of LeV in VI-CK and the STTM transformants treated by 25°C (E) and 37°C (F), respectively. ns represents no significant differences; ** represents extremely significant difference, *P* < 0.01.

RT-qPCR analysis revealed that both *Pre-milR21* and *led-milR-21* exhibited significantly elevated expression levels in the HS-treated groups (VI-CK_37, OEPre_37, and STTM_37) compared to their 25°C counterparts. Notably, the expression levels of these two RNA molecules in *Pre-milR21*-overexpressing transformants (OEPre_37) were significantly higher than in both the empty vector control (VI-CK_37) and the *led-milR-21*-silenced transformants (STTM_37) (Fig. 6A and B). Compared to VI-CK_37, the expression of two LeV ORFs was markedly upregulated in OEPre_37 and downregulated in STTM_37 (Fig. 6C through F). Similarly, the expression of *LeDCL1* in OEPre_37 mycelia was significantly higher than in both VI-CK_25 and VI-CK_37, whereas STTM_37 showed significantly lower *LeDCL1* expression compared to the same controls (Fig. S11). These results suggest that HS modulates a regulatory network involving LeV replication, the biosynthesis of *led-milR-21*, and *LeDCL1* activity.

**Fig 7:**
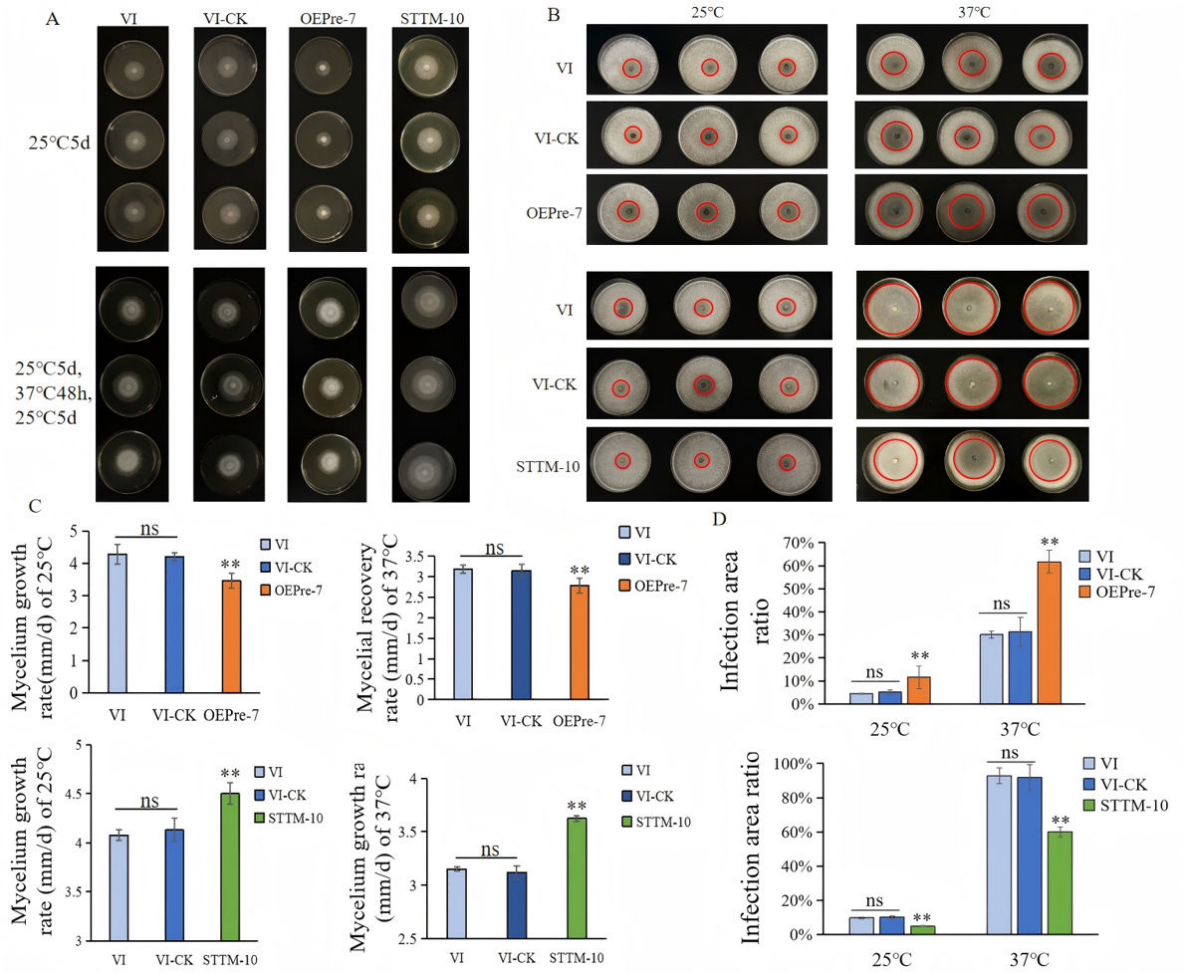
The results of mycelial growth rate, the ability of thermotolerance, and the mycelial resistance to *Trichoderma atroviride* of the OEPre and SMMT transformants and the control strains. (A) The colonies’ phenotypic characteristics under different temperature treatments. (B) In the 25°C group, the mycelial blocks were cultured at 25°C for 10 d, and then were removed, and a *T. viride* mycelial block was finally inoculated in the center of the plate; in the 37°C group, the mycelial blocks were cultured at 25°C for 8 d, treated at 37°C for 48 h, and then were removed, and a *T. viride* mycelial block was finally inoculated in the center of the plate. The red circle represents the area infected by *T. Viride*. (C) The growth rate of OEPre and STTM transformants and the control strains under different temperature treatments. (D) Proportion of colonial areas infected by *T. viride*. ns represents no significant difference; ** represents extremely significant difference, *P* < 0.01.

Using VI-CK as a reference, five transformants with the highest *Pre-milR21* expression levels (OEPre) and five with the lowest levels (STTM) were selected to assess mycelial growth rate and thermotolerance in *L. edodes*. The results demonstrated a negative correlation between *Pre-milR21*/*led-milR-21* expression levels and fungal fitness: overexpression of *Pre-milR21* significantly inhibited both thermotolerance and mycelial growth, whereas silencing (STTM) enhanced these traits (Fig. 7; Fig. S12). Our previous study demonstrated that LeV infection reduces the resistance of *L. edodes* mycelia to the fungal antagonist *Trichoderma atroviride* following HS (37). This study not only corroborates those findings but also identifies *led-milR-21* as a key mediator of this LeV-driven susceptibility.

### Molecular regulatory network of milRNA (*led-milR-21*) affecting thermotolerance in *Lentinula edodes*

**Fig 8:**
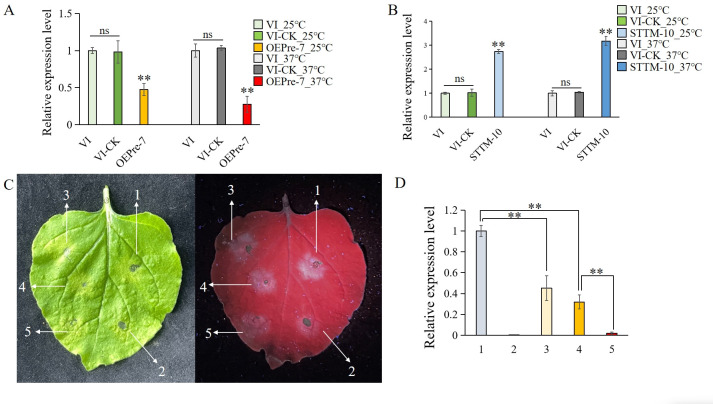
Verification of the target gene *LE01Gene01783* of led-milR-21. (A) RT-qPCR analysis of the expression levels of *LE01Gene01783* in OEPre transformants. (B) RT-qPCR analysis of the expression levels of *LE01Gene01783* in STTM transformants. (C) The results of tobacco transient expression tests. Note: 1-0.3 OD a + 0.3 OD c, 2-0.3 OD b + 0.3 OD c, 3-0.1 OD a + 0.3 OD b, 4-0.3 OD a + 0.3 OD b, 5-0.5 OD a + 0.3 OD b; the numerical value from the OD represents the OD_600_ value, “a” presents the *Agrobacterium* containing recombinant plasmid P1391-3' UTR1783-GFP, “b” presents the *Agrobacterium* containing recombinant plasmid P1300-35S-PremilR21, “c” presents the *Agrobacterium* containing no-load P1300-35S. (D) RT-qPCR analysis of GFP expression levels in the different parts of leaves. Numbers on the abscissa represent the above-mentioned in C.

We identified 46 candidate target genes for led-milR-21 based on the 3′-UTR sequences within the *L. edodes* genome. Notably, three genes (*LE01Gene01783*, *LE01Gene03491*, and *LE01Gene06644*) were significantly upregulated (*P* < 0.05) in VF following HS treatment. Among these, LE01Gene01783—a transcription factor harboring a CCAAT-box and a conserved CBFB/NFYA domain (Pfam ID: PF02045)—was previously reported to exhibit a 96.34-fold upregulation in a heat-susceptible *L. edodes* strain post-HS (8). Our study further demonstrated that the expression levels of *LE01Gene01783* were significantly modulated in OEPre and STTM transformants compared to VI-CK (Fig. 8A and B).

To further validate the interaction between led-milR-21 and the 3′ UTR region of LE01Gene01783, a tobacco transient fluorescence expression system was employed. The positive control consisted of leaves co-infiltrated with an equal mixture of *Agrobacterium* A (harboring the recombinant plasmid P1391-3′ UTR1783-GFP) and *Agrobacterium* C (empty vector P1300-35S), which exhibited strong GFP fluorescence. The negative control involved leaves co-infiltrated with *Agrobacterium* B (carrying P1300-35S-PremilR21) and *Agrobacterium* C, showing minimal GFP fluorescence. For experimental treatments, three concentrations of *Agrobacterium* B (0.1 OD, 0.3 OD, and 0.5 OD, respectively) were separately mixed with the concentration of 0.3 OD *Agrobacterium* A for leaf infiltration. Ultraviolet observation at 3 days post-infiltration revealed intense fluorescence in the positive control (Fig. 8C, arrow 1) and negligible fluorescence in the negative control (Fig. 8C, arrow 2). All treatment groups displayed significantly weaker fluorescence than the positive control (Fig. 8C, arrows 3, 4, and 5). Notably, leaves infiltrated with 0.5 OD A + 0.3 OD B (Fig. 8C, arrow 5) showed no detectable fluorescence. RT-qPCR analysis further demonstrated that GFP expression levels in all treatment groups were significantly lower than in the positive control. Moreover, the high-concentration treatment (0.5 OD A + 0.3 OD B) exhibited significantly reduced GFP expression compared to medium- and low-concentration treatments (Fig. 8D). These findings underscored LE01Gene01783 as a pivotal target gene of led-milR-21.

To further investigate the possible molecular mechanism regulated by led-milR-21, RNA-seq data from three *Pre-milR21*-overexpressing transformants (OEPre_2, OEPre_4, and OEPre_7) and three empty vector control strains (VI-CK_1, VI-CK_2, and VI-CK_3) were further obtained (BioProject accession PRJNA1124433). Sequencing statistics are detailed in Table S4, and the PCA analysis result is presented in Fig. S13. Comparative analysis between OEPre_37 and VI-CK_37 identified 3,166 DEGs, with 1,712 upregulated and 1,454 downregulated in response to HS. Among these DEGs, 23 HSPs (5 upregulated, 17 downregulated) and 32 TFs (13 upregulated, 19 downregulated) exhibited differential expression (Fig. S14; Table S5), suggesting impaired HS adaptation in OEPre_37 compared to VI-CK_37. KEGG enrichment analysis revealed that the 1,712 upregulated DEGs in OEPre_37 were enriched in five pathways: aminoacyl-tRNA biosynthesis, folate-mediated one-carbon metabolism, nucleocytoplasmic transport, ribosome biogenesis, and secondary metabolite biosynthesis (Fig. S15A; Table S6). Conversely, 1,454 downregulated DEGs were associated with nine pathways, including arginine and proline metabolism, branched-chain amino acid degradation, endoplasmic reticulum protein processing, tryptophan metabolism, and fatty acid degradation (Fig. S15B to S17; Table S6). These pathways overlap with those potentially regulated by LE01Gene01783 and align with the thermotolerance mechanisms reported in *L. edodes* (8). Specifically, the tryptophan metabolic pathway, previously linked to fungal thermotolerance by Wang et al. (2018) (8), was prominently affected.

## DISCUSSION

Our study is the first to establish the impact of high temperature on the interaction between mycoviruses and mushrooms. When mycelia fully grown at 25°C undergo a 37°C heat shock, the LeV-host equilibrium is disrupted, resulting in enhanced LeV replication (Fig. 1C) and reduced thermotolerance in virus-infected (VI) *L. edodes* (Fig. 1D). These changes lead to symptoms including decreased mycelial growth, impaired hyphal aggregation, and abnormal browning (Fig. 1E).

Higher temperatures can greatly increase the susceptibility of uninfected hosts like tobacco to viruses; for instance, tobacco is much more susceptible to *Tobacco mosaic virus* at 36°C than at 20°C (38). However, in infected organisms, higher temperatures may alter virus-host interactions, typically significantly restrict viral spread and replication, and induce latent symptoms or reduce viruses’ pathogenicity (39). Elevated temperature is also reported to suppress a range of antiviral responses and be associated with more severe symptoms (40–42). Previous research on the impact of higher temperatures on virus-host interactions in fungi is limited. The fungus *Curvularia protuberata* carries *Curvularia thermal tolerance virus* and develops a three-way symbiotic relationship with plants to enable their survival in extreme soil temperatures (13). In other references, mycoviruses such as *Heterobasidion RNA virus 3* (HetRV3-ec1) and *Heterobasidion RNA virus 6* (HetRV3-ec1) were reported to manipulate the physiology of each particular host species in a temperature-dependent manner, demonstrating negative or positive effects on their hosts under higher temperature (19, 20). However, the mechanisms underlying temperature-dependent modulation of virus-host interactions remain poorly understood. In this study, *L. edodes* showed limited activation of RNAi defenses against LeV infection under normal temperature conditions. By contrast, exposure to 37°C for 24 or 48 h markedly enhanced LeV accumulation, upregulated key RNAi pathway components (e.g., the *LeDCL1* gene), and increased expression of a host-encoded microRNA (led-milRNA-21). Functional characterization of *led-milRNA-21* revealed that its expression level positively correlates with both LeV accumulation and *LeDCL1* transcription (Fig. 6). Furthermore, this microRNA negatively regulates mycelial growth, heat tolerance, and resistance to *T. atroviride* (Fig. 7). Collectively, these findings suggest that LeV may exploit *led-milRNA-21* as a regulatory hub to suppress host growth and thermotolerance under HS conditions.

**Fig 9:**
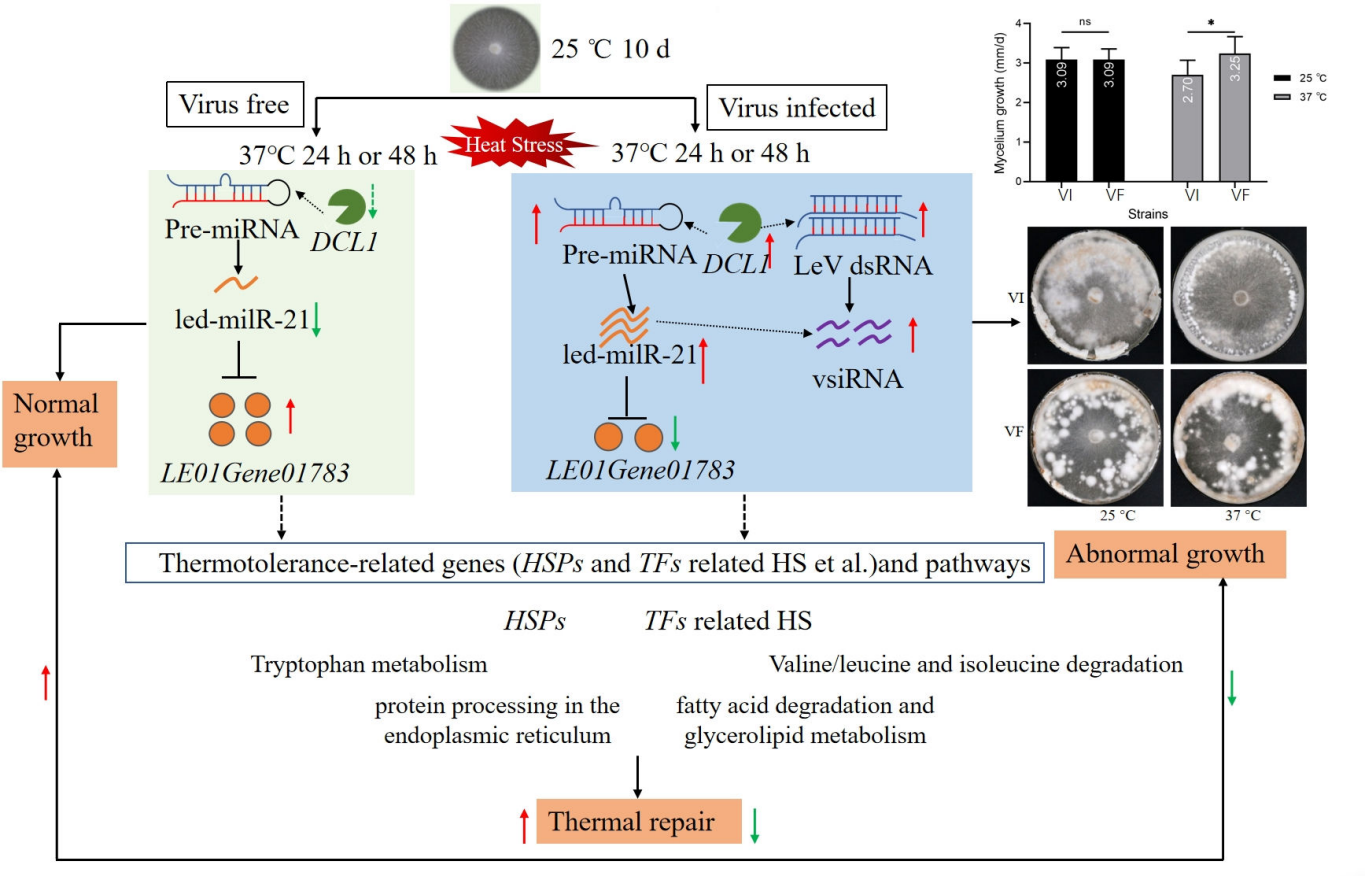
A proposal model for interactions between LeV and *Lentinula edodes* after HS through interfering with led-milR21 expression. The red arrow represents upregulation, the green arrow represents downregulation, and the dashed arrow indicates that it was possibly related to the function.

To our knowledge, this is the first report of a fungal miRNA (led-milR-21) induced by both a mycovirus and HS. This discovery establishes a framework for investigating the tripartite interaction among thermal stress, LeV, and *L. edodes*. However, key mechanisms remain unresolved, including (i) pathways driving LeV replication and host antiviral responses under HS; (ii) regulatory networks controlling the biogenesis of *led-milR-21* during heat-virus co-stress; and (iii) molecular links between *led-milR-21* and metabolic reprogramming. Our research revealed that led-milR-21 targets *LE01Gene01783*, which perturbs multiple pathways, including tryptophan metabolism, arginine and proline metabolism, valine, leucine, and isoleucine degradation, protein processing in the endoplasmic reticulum, fatty acid degradation, glycerolipid metabolism, etc. (Fig. 9; Fig. S15 to S17). Nevertheless, whether *led-milR-21* targets additional genes and how its regulatory network integrates with LeV virulence require further exploration.

### Conclusion

This study demonstrates how elevated temperatures alter the interaction between LeV and *L. edodes*, triggering a transition from cryptic to symptomatic viral infection. Under normal temperatures (25°C), *led-milR-21* expression remains low in the VF, while the thermotolerance-associated transcription factors such as *LE01Gene01783* and other HS response genes (e.g., *HSPs*) are significantly upregulated. This transcriptional activation possibly modulates HS-related metabolic pathways, including tryptophan metabolism, amino acid metabolism, endoplasmic reticulum protein processing, fatty acid degradation, glycerolipid metabolism, etc., which collectively counteract HS-induced cellular damage (Fig. 9).

In VI under 25°C, LeV mRNA levels remain suppressed, maintaining a balanced virus-host interaction. However, when fully colonized mycelia are subjected to HS (37°C for 48 h), LeV replication surges, leading to *DCL1*-mediated degradation of LeV genomic RNA. Concurrently, *LeDCL1* and other RNA interference (RNAi) pathway genes (e.g., *LeAGO8*, *LeRDR1*, *LeRDR5*, *LeRDR6*) are recruited during the *led-milR-21* biogenesis, amplifying its production. Consequently, *led-milR-21*-dependent silencing of *LE01Gene01783* occurs upon LeV infection, diminishing the heat repair capacity. This results in a reduced mycelial growth rate and decreased resistance of VI mycelia to *T. atroviride*, as well as influencing hyphal aggregation and browning in the subsequent cultures (Fig. 9).

## MATERIALS AND METHODS

### Fungal strains

The fungal strains used in this study included two *L. edodes* strains, SY1 (LeV-infected, VI) and SY1-R8 (LeV-free, VF), and a *Trichoderma atroviride* strain, 92-1. SY1 was a cultivated strain used in field cultivation in China (Fig. S18A). The SY1-R8 was obtained via ribavirin detoxification of SY1 (Fig. S18B). According to the industrial standard “Identification of Edible Mushroom Strains by ISSR Method” (NY/T 1730-2009), 10 ISSR primers were randomly selected to analyze the molecular fingerprints for the VI and VF strains (Table S7), yielding identical ISSR band patterns (Fig. S19C), indicating that ribavirin treatment did not significantly affect the *L. edodes* strain’s genetic information. The *Trichoderma atroviride* strain 92-1, identified from diseased *L. edodes* samples, was obtained in our previous research (1, 37).

### Heat stress treatment

*L. edodes* mycelia were grown on an MYG solid medium at 25°C in darkness for 2 d until germination and transferred to a fresh plate for further culturing at 19°C, 22°C, 25°C, 28°C, 31°C, and 34°C, respectively. The growth rate of the mycelia was measured using the cross-line method. After a 7-day culturing, the mycelia were collected, flash-frozen in liquid nitrogen, and stored at −80°C for subsequent viral nucleic acid quantification. For heat stress assays, *L. edodes* mycelia were grown on MYG medium for 10 d, reaching full plate coverage, and they were then exposed to 25°C, 37°C, 40°C, and 42°C for 24 h, respectively. After HS treatment, the mycelia were immediately collected, flash-frozen in liquid nitrogen, and stored at −80°C for viral nucleic acid quantification. The mycelial blocks from colony edges were transferred to fresh MYG medium and incubated at 25°C in darkness for growth rate measurement using the cross-line method. The residual mycelia on the plates were returned to a 5-day dark incubation at 25°C and subsequently subjected to a regimen of alternating 12 hour periods of light exposure at 300 Lux and darkness to observe the changes in phenotypic expression of the colonies.

### RNA extraction, RT-PCR, and RT-qPCR

Total RNA from *L. edodes* mycelia was extracted using RNAiso Plus (TaKaRa) and reverse-transcribed using the HiScript II Q RT SuperMix for qPCR (+gDNA wiper) kit (TaKaRa). The PCR cycle was as follows: 95°C for 5 min, followed by 34 cycles of 95°C for 30 s, 55°C for 30 s, and 72°C for 30 s, with a final extension at 72°C for 10 min. PCR products were analyzed by 1% agarose gel electrophoresis and stained with ethidium bromide. For quantification of relative gene expression levels in *L. edodes* strains, RT-qPCR was performed with the *lectin gene of L. edodes (Leactin*) as the reference gene. The 10 µL RT-qPCR mixture included 1 µL cDNA, 5 µL AceQTM qPCR SYBR Green Master Mix, 0.5 µL of each primer (10 µM), and 3 µL ddH2O. The two-step amplification included a pre-denaturation at 95°C for 5 min, followed by 40 cycles of 95°C for 10 s and 60°C for 30 s; and a final melting curve analysis was conducted from 65°C to 95°C, increasing by 0.5°C increments, with fluorescence signals collected every 5 seconds. Relative gene expression levels were calculated using the 2^−ΔΔCT^ method. The primers used for RT-PCR and RT-qPCR are listed in Table S8.

### Microscopic structure observation

*L. edodes* were grown on MYG plates with cellophane, and the mycelial blocks were inoculated in the center. Once the mycelia covered half the plate, 4 mm^2^ pieces of tissues containing the mycelial tips area were excised with sharp tools and immediately immersed in 0.1 mol/L phosphate-buffered saline (PBS) containing 2.5% (vol/vol) glutaraldehyde. After a 30 minute treatment for full submersion, the samples were prepared for SEM and TEM according to the methods described by Mubshar et al. (43). The samples were then examined using a SEM (JSM-6390LV, JEOL) and TEM (H-7650, Hitachi), with observation results captured by a CCD camera (Model 832ORIUS, Gatan).

### Transcriptomic analysis for the LeV and HS co-influence on thermotolerance-related gene expression in *L. edodes*

The VI and VF strains were cultured on MYG plates with cellophane at 25°C in darkness for 10 d, followed by a heat shock (HS) at 37°C for 48 h. A control group was treated at 25°C for the same duration. Samples were collected at two time points: (i) T1 immediately post-HS, flash-frozen in liquid nitrogen, and stored at −80°C; (ii) T2—after a 5-day post-HS recovery at 25°C in darkness, followed by 10 d of 12 h:12 h light (300 Lux)—dark cycle, then similarly processed. Each time point had four samples: control (25VI and 25VF) and treatment (37VI and 37VF), with three biological replicates per sample. Total RNA extraction, mRNA purification, strand-specific cDNA library construction, and sequencing were conducted by BGI (Shenzhen) using the Illumina HiSeqTM 4000 platform, with a paired-end read length of 150 bp, yielding at least 6 G of clean data per sample. Raw transcriptome sequencing data were filtered using Trimmomatic-0.33 to extract clean reads, which were then mapped to the *L. edodes* W1-26 reference genome using Hisat2 (https://mycocosm.jgi.doe.gov/Lentinedodes1/Lentinedodes1.home.html) (44). Gene read counts aligned to *L. edodes* genes were calculated using HTSeq (45), and gene expression levels were normalized to TPM (Transcripts Per Million) using TBtools (46). Differential expression was assessed using EdgeR (47), identifying genes with a false discovery rate (FDR) of ≤0.01 and a fold change magnitude of ≥1. Expression trend of DEGs was analyzed using STEM v1.3.11 with default threshold settings, and gene ontology (GO) enrichment analysis was performed using clusterProfiler with an FDR of <0.05 as the significance threshold. Analyses of DEGs based on GO and KEGG enrichment were conducted using TBtools and online tools (https://www.omicshare.com/tools/Home/Soft/getsoft), applying a *P*-value filter of ≤0.05 (45, 48, 49). The results were visualized with online tools WEGO (https://wego.genomics.cn/) and the R package ggplot2. Weighted gene co-expression network analysis (WGCNA) (36) was performed with a coefficient of variation >0.15 in the TPM expression matrix, correlating with HS treatment, LeV infection, and sampling time. Mycelial traits were used to identify modules correlated with these attributes, with a soft threshold of 7 and a correlation threshold of |r| > 0.3, *P* < 0.05. Mycelial vitality (mycelial recovery growth rate and *Trichoderma* resistance after heat stress) and colony browning degree were used as traits to identify modules correlated with these two target attributes, with a soft threshold of 7, and a correlation threshold of |r| > 0.3 and *P* < 0.05.

### Small RNA sequencing and analysis

The transcriptome samples (control: 25VI and 25VF; treatment: 37VI and 37VF) were also subjected to small RNA (sRNA) sequencing. Total RNA extraction, sRNA purification, and library construction were performed by BGI (Shenzhen), yielding 20 M clean tags per sample using the BGISEQ-500 platform after an 18-30 nt sRNA size selection by SDS-PAGE separation. Using the sports software (50), redundant and unique tags of 18-30 nt were extracted from the clean tags of each sRNA library and aligned to the genome of the *L. edodes* monokaryotic strain W1-26 using bowtie2, without allowing gaps or mismatches. Annotation and classification of sRNAs such as rRNA, tRNA, snRNA, and snoRNA were performed using the Rfam 14.2 database (51), and milRNA prediction was conducted using the miR-PREFeR software (52). miR-PREFeR was used to predict milRNAs, adjusting parameters to PRECURSOR_LEN = 150 (maximum length of precursor allowed), READS_DEPTH_CUTOFF = 10, MIN_MATURE_LEN = 18, MAX_MATURE_LEN = 30, with others default (65). The identified milRNAs were then aligned to the miRbase 22 database using bowtie2, allowing two mismatches, to differentiate known from novel milRNAs (53). Given the limited fungal miRNAs in miRbase, a curated database of 663 known fungal milRNAs from the literature was used for further alignment (Table S9), also allowing two mismatches, to identify both known and novel fungal milRNAs. MilRNA expression levels were quantified using the miR-PREFeR software, with normalization to CPM (counts per million), and the raw counts matrix was converted to a CPM matrix using 18-30 nt clean redundant tags. The snRNA U6 from *L. edodes* was used as the reference gene (54), and the top three differentially expressed milRNAs were validated by stem-loop RT-qPCR. The miRNA from each *L. edodes* sample was purified using the miRcute miRNA Extraction and Separation Kit (Beijing Tiangen Biotech). cDNA was synthesized using the miRNA 1st Strand cDNA Synthesis Kit (by stem-loop) (Vazyme), employing random hexamer primers for the U6 gene and specific stem-loop primers for milRNAs. qPCR was conducted using the miRNA Universal SYBR qPCR Master Mix (Vazyme), with primers detailed in Table S10. Target gene prediction for *L. edodes* milRNAs was conducted following the method used for the model mushroom *Coprinopsis cinerea* (55, 56), extracting the 3′ UTR sequences of all coding genes from the W1-26 genome were extracted, and target genes were predicted using miRanda software (57), using a score threshold >150.

### Functional analysis of led-milR-21

The precursor gene of led-milR-21, Pre-milR21, was selected to construct an overexpression vector. The STTM-milR21 sequence, synthesized by Wuhan Tianyi Huiyuan Company, was employed with the short tandem target mimic (STTM) technique to develop the silencing vector for led-milR-21 (58). Both vectors were constructed using the pCAMBIA1300-g plasmid, with target gene and linearized plasmid fragments joined by homologous recombination. The recombinant plasmids, confirmed by sequencing, were then transformed into *Agrobacterium* and subsequently into VI strains. The vector construction outcomes are shown in Fig. S19 and S20, with the primer information provided in Table S11. Growth rate and HS tests were conducted as aforementioned. The resistance of the obtained positive transformants to *T. atroviride* was evaluated by inoculating mycelial blocks onto MYG plates and incubating at 25°C in the dark until the mycelia fully covered the plates. Five plates were then treated at 37°C in the dark for 48 h, while the remaining five plates were kept at 25°C for the same duration. After that, the *L. edodes* blocks were removed from plates, and *T. atroviride* mycelial blocks were inoculated at the same site. All treatments were incubated at 25°C. The *L. edodes* colony browning area coverage post-*T. atroviride* inoculation was uniformly analyzed and measured using ImageJ 1.52a software. The browning ratio of the *L. edodes* colony, calculated as “browning area/plate area,” was used to assess the resistance of *L. edodes* strains, with a lower ratio indicating stronger resistance to *T. atroviride*.

### Construction of a new LeV inoculate carrying the hygromycin (*Hyg*) resistance gene

The plasmid pCAMBIA1300-g carrying the Hyg resistance gene was introduced into *A. tumefaciens* and used to infect the VF strain, yielding a new VF strain with Hyg resistance (VF-Hyg). This strain was cultured at 25°C and 37°C for 48 hours, with VF as a control, to verify the effect of *Hyg* introduction on VF. Three methods were employed to test LeV’s horizontal transmission: co-culturing VI and VF-Hyg on the same plate for 5 d; pre-culturing VI for 3 d, then VF-Hyg for 2 d; and pre-culturing VF-Hyg for 3 d, then VI for 2 d. After contact, mycelial blocks from the VF-Hyg area distant from VI were transferred to a fresh MYG medium containing 4 µg/mL hygromycin. RT-PCR was performed to analyze the *Hyg* gene in VF-Hyg and VF-Hyg + virus strains, as well as the *RdRp* gene of LeV. RT-qPCR was conducted to confirm the presence of *Hyg* and viral ORFs in VF-Hyg and VF-Hyg + virus strains. The primers used are detailed in Table S12.

### Validation of the *LE01Gene01783* gene targeted by led-milR-21

Using *Leactin* as the reference gene, RT-qPCR was conducted to measure the relative expression level of LE01Gene01783 in *L. edodes* transformants as aforementioned, with primers listed in Table S13. The P1391-eGFP plasmid was digested with restriction enzymes *Bam*HI and *Kpn*I, and then combined with a 212 bp sequence flanking the 3′ UTR region of LE01Gene01783 interacting with led-milR-21, yielding a recombinant plasmid, P1391-1783-eGFP. The plasmid P1300-35S was similarly digested with restriction enzymes *Sal*I and *Kpn*I to incorporate the *pre-milR21* sequence, yielding a recombinant plasmid, P1300-35S-premilR21. The primer information used is listed in Table S13, and plasmid maps and validation are shown in Fig. S21. The inhibitory effect of led-milR-21 on LE01Gene01783 was tested in *Nicotiana benthamiana* using an Agrobacterium-mediated transient expression system. After 3 d, GFP fluorescence in the injected leaf sections was visualized under UV light at 365 nm, and GFP expression on the injection sites was analyzed by qRT-qPCR analysis, using primers listed in Table S13.

### Transcriptome analysis of the led-milR-21 overexpression transformants

The VI-CK and overexpression transformants (OEPre2, OEPre4, and OEPre7) were cultured on MYG plates with cellophane at 25°C in darkness for 10 d. They were then exposed to two temperature treatments: 37°C for 48 h (VICK_37 and OEPre_37) and 25°C for 48 h (VICK_25 and OEPre_25). After treatment, the mycelia from both control (VICK_25 and VICK_37) and the treatment groups (OEPre_25 and OEPre_37) were harvested, flash-frozen in liquid nitrogen, and stored at −80°C. Total RNA extraction, mRNA purification, strand-specific cDNA library construction, and sequencing were conducted by Frasergen (Wuhan) using the Illumina 1.9 platform, yielding at least 4 G of raw paired-end 150 bp reads per sample. Sequencing quality control and differential gene screening adhered to the criteria in section 2.5. GO and KEGG enrichment analyses of the differentially expressed genes were performed using an online tool (https://www.omicshare.com/tools/Home/Soft/getsoft), applying a *P* value threshold of ≤0.05 (46).

## Data Availability

The raw sequencing data supporting this study are openly available in the NCBI Sequence Read Archive (SRA) under accession numbers PRJNA1120119 and PRJNA1124433.

## SUPPLEMENTAL MATERIAL

Supplemental figures jvi.00084-25-s0001.pdf Figures S1 to S21.

Supplemental tables jvi.00084-25-s0002.xlsx Tables S1 to S13.

ASM does not own the copyrights to Supplemental Material that may be linked to, or accessed through, an article. The authors have granted ASM a non-exclusive, world-wide license to publish the Supplemental Material files. Please contact the corresponding author directly for reuse.
