# Supplementary material for: New insights into temperature-impacted mycovirus-fungus interactions regulated by a microRNA in Lentinula edodes
Source: J Virol. 2025 Aug 20;99(9):e00084-25. doi: 10.1128/jvi.00084-25 (PMC12456130; doi:10.1128/jvi.00084-25)
Supplement: Supplemental figures — Figures S1 to S21. [file jvi.00084-25-s0001.pdf]

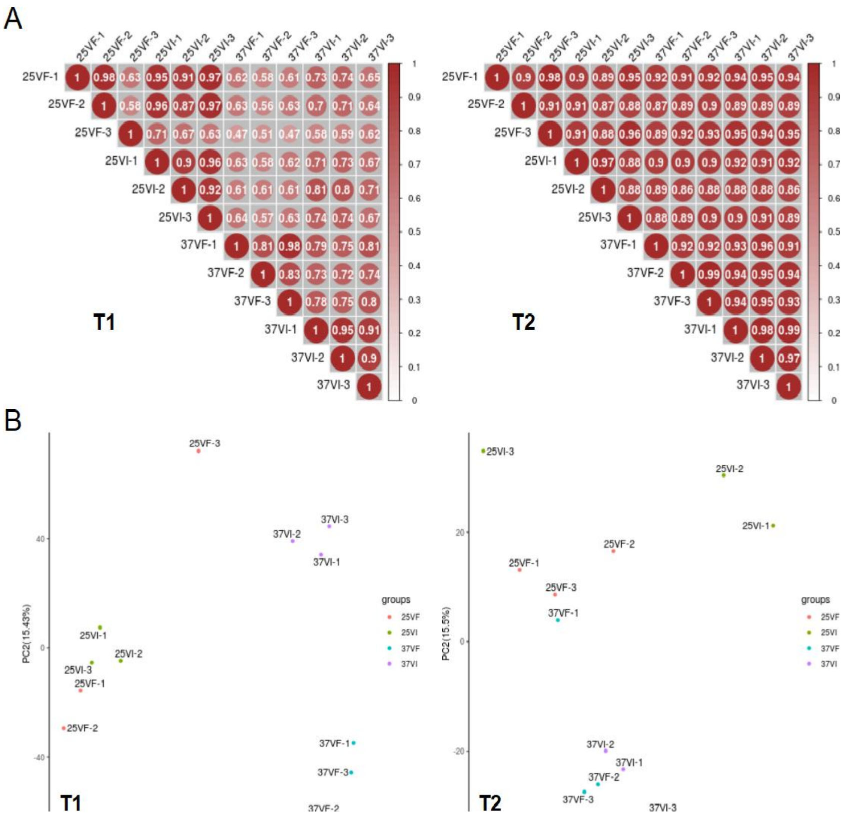

Fig.S1. Correlation coefficient and principal component analyses of the sequenced VI and VF stains. A. The results of correlation coefficient analysis; B. The results of principal component analysis. Note: -1, -2, -3 mean three biological replicates; T1- the mycelia were collected immediately post-HS and sequenced; T2 - HS treatment and then after a 5-day post-HS recovery at 25°C in darkness, followed by a 10-d of 12h:12h light (300 Lux)-dark cycle, then the mycelia were collected and sequenced.

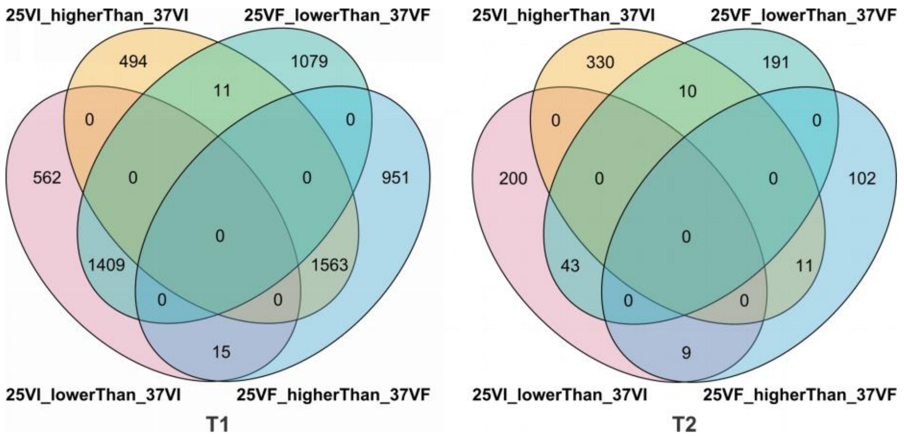

Fig.S2. Venn diagrams of differential expressed genes of VI and VF after heat stress of 37 °C for 48 h. Note: T1- the mycelia were collected immediately post-HS and sequenced; T2 - HS treatment and then after a 5-day post-HS recovery at 25°C in darkness, followed by a 10-d of 12h:12h light (300 Lux)-dark cycle, then the mycelia were collected and sequenced.

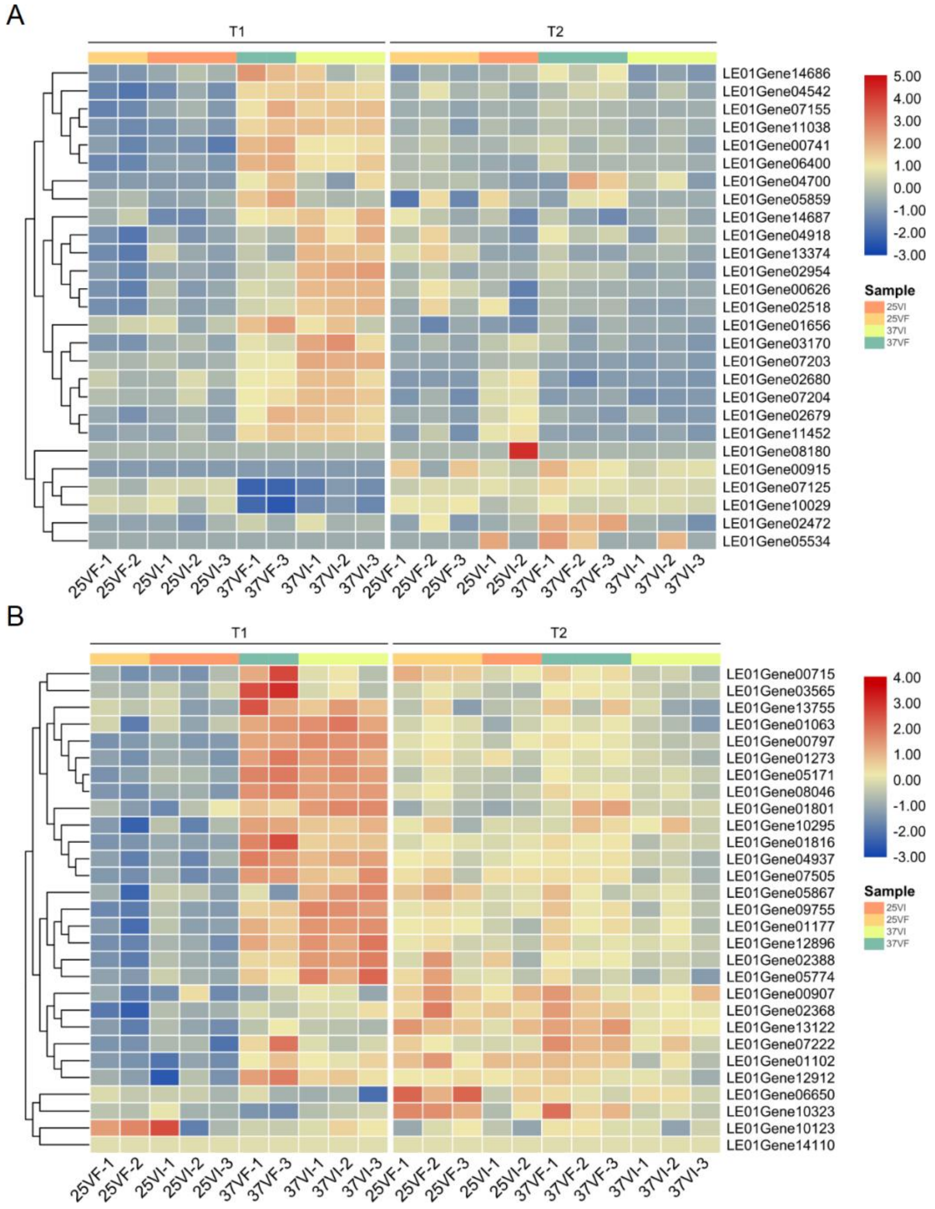

Fig.S3. The heat map of differential expressed heat shock response related genes (*HSPs*). Note: T1- the mycelia were collected immediately post-HS and sequenced; T2 - HS treatment and then after a 5-day post-HS recovery at 25°C in darkness, followed by a 10-d of 12h:12h light (300 Lux)-dark cycle, then the mycelia were collected and sequenced.

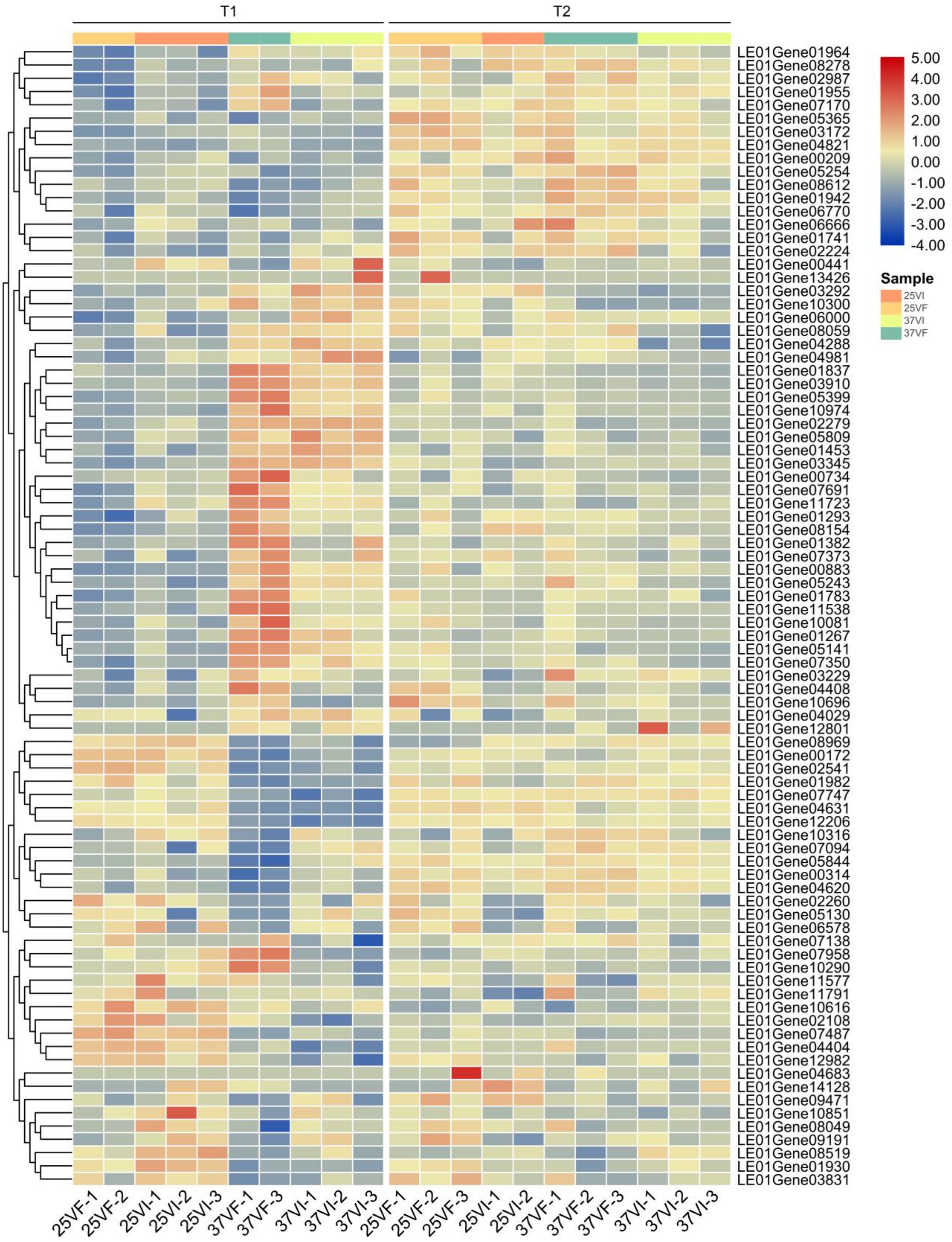

Fig.S4. The heat map of differential expressed heat shock response related transcription factors (*HSFs*). Note: T1- the mycelia were collected immediately post-HS and sequenced; T2 - HS treatment and then after a 5-day post-HS recovery at 25°C in darkness, followed by a 10-d of 12h:12h light (300 Lux)-dark cycle, then the mycelia were collected and sequenced.

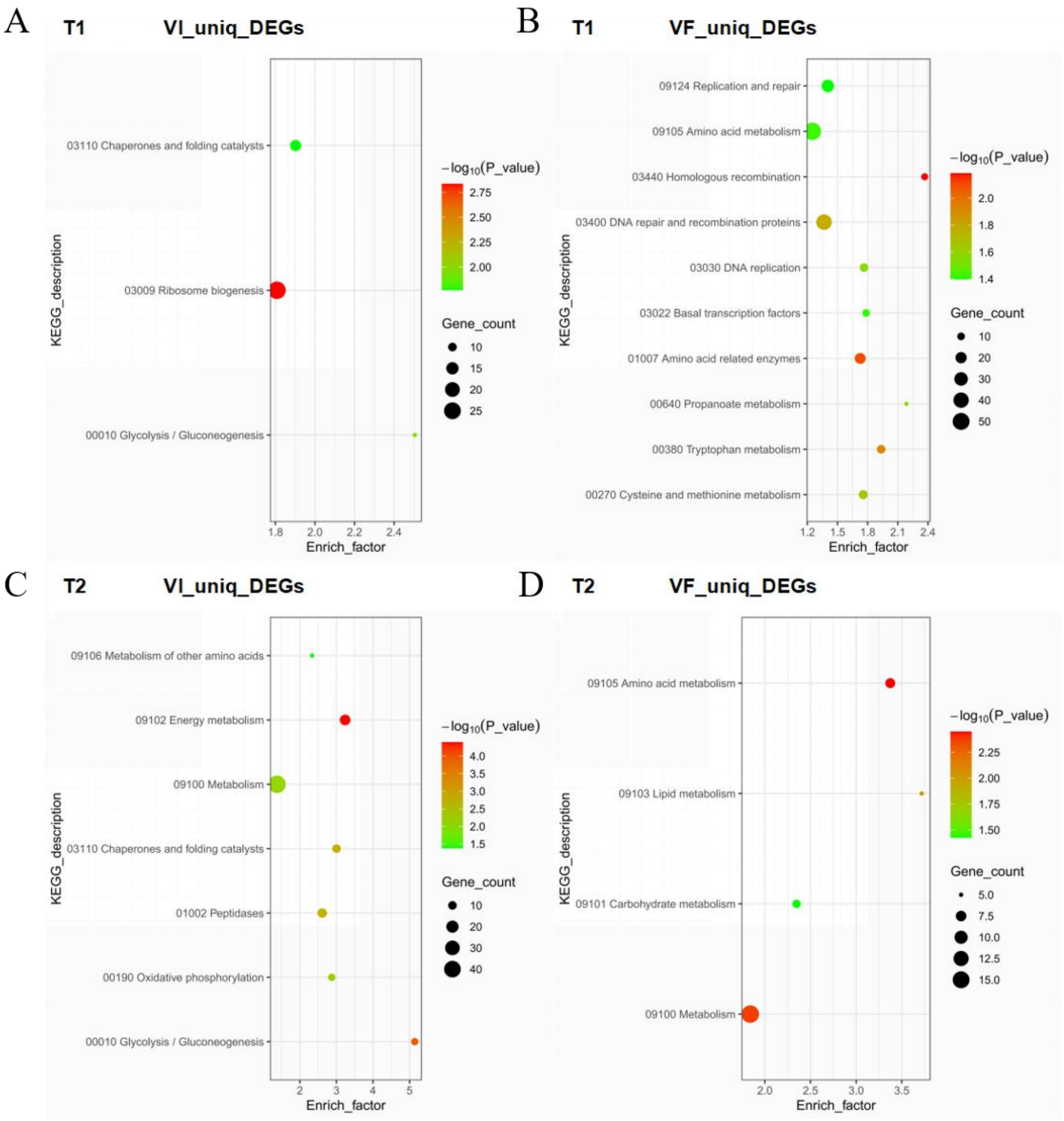

Fig. S5. The top KEGG enrichment pathways of DEGs after heat stress of 37 °C for 48 h at T1 stage and T2 stage. A. The top KEGG enrichment pathways of VI at T1 stage; B. The top KEGG enrichment pathways of VF at T1 stage; C. The top KEGG enrichment pathways of VI at T2 stage; D. The top KEGG enrichment pathways of VF at T2 stage. Note: T1- the mycelia were collected immediately post-HS and sequenced; T2 - HS treatment and then after a 5-day post-HS recovery at 25°C in darkness, followed by a 10-d of 12h:12h light (300 Lux)-dark cycle, then the mycelia were collected and sequenced.

A

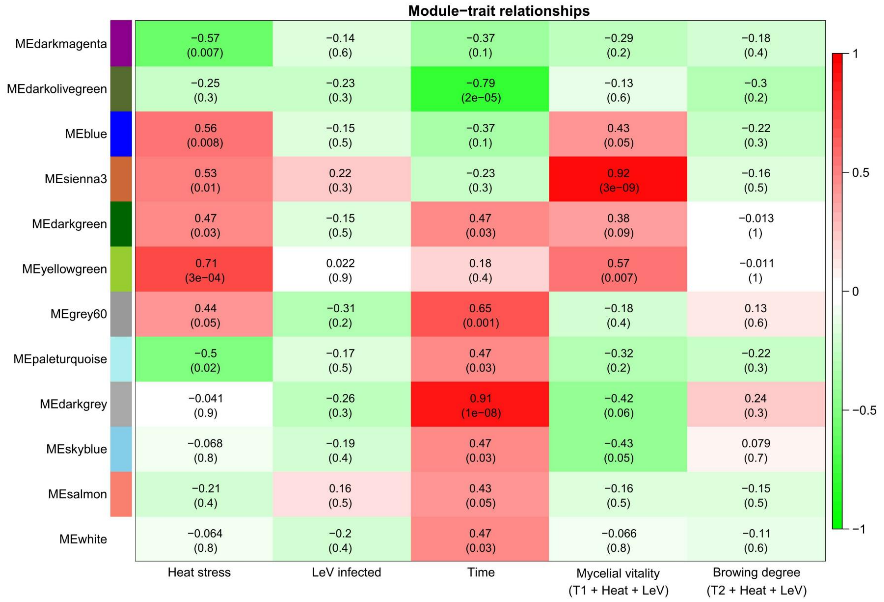

B

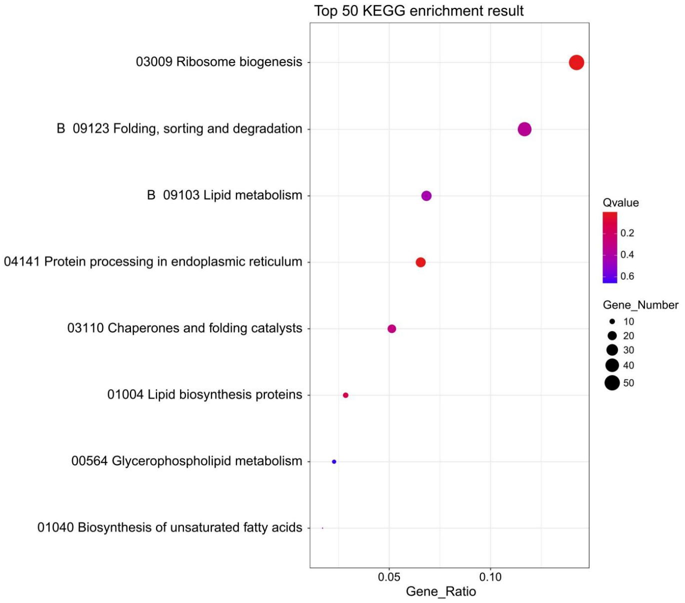

Fig.S6. Weighted gene co-expression network analysis (WGCNA) analysis results of correlation between gene modules and different sample attributes (A) and the top enriched KEGG pathways in module MEsienna3 related to both HS treatment and LeV infection (B). Note: WGCNA was performed with a coefficient of variation > 0.15 in the TPM expression matrix, correlating with HS treatment, LeV infection, and sampling time. Mycelial traits were used to identify modules correlated with these attributes, with a soft threshold of 7 and a correlation threshold of $|r| > 0.3$, $p < 0.05$. Mycelial vitality (mycelial recovery growth rate and *Trichoderma* resistance after heat stress) and colony browning degree were used as traits to identify modules correlated with these two target attributes, with a soft threshold of 7, and a correlation threshold of $|r| > 0.3$ and $p < 0.05$.

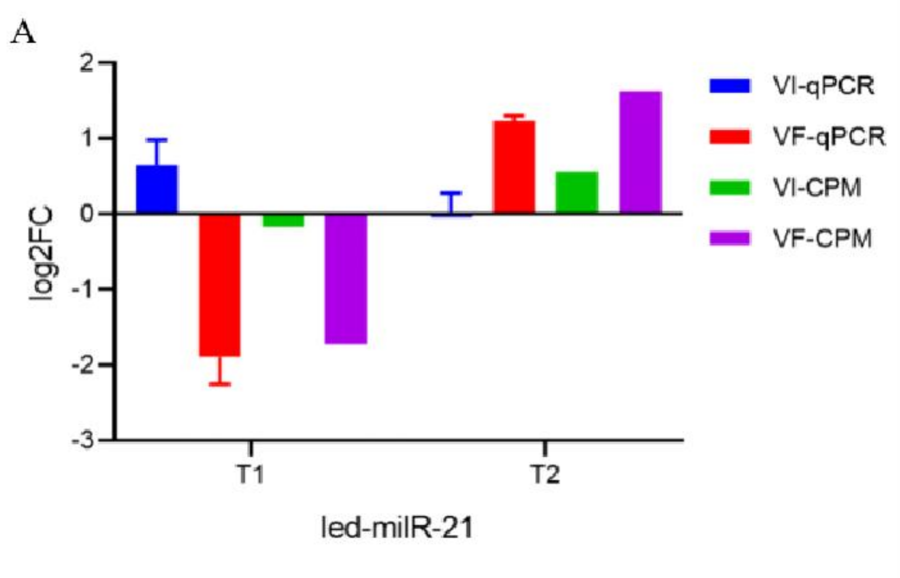

Fig.S7. The result of expression level of led-milR-21 after heat stress of 37 °C for 48 h by stem-loop RT-qPCR and analysis of the CPM values. Note: T1- the mycelia were collected immediately post-HS and sequenced; T2 - HS treatment and then after a 5-day post-HS recovery at 25°C in darkness, followed by a 10-d of 12h:12h light (300 Lux)-dark cycle, then the mycelia were collected and sequenced.

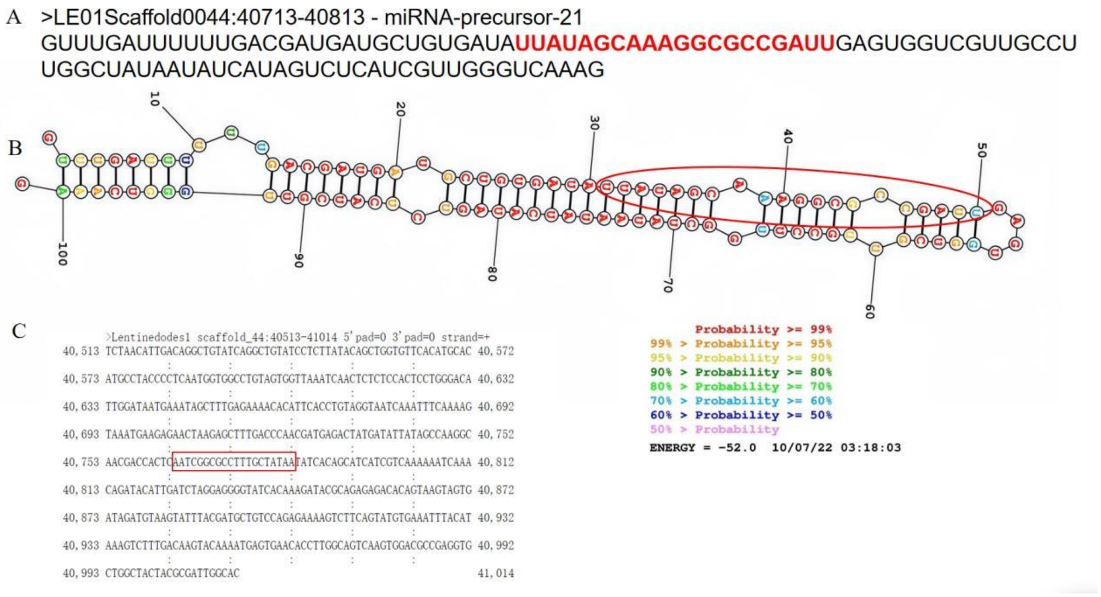

Fig.S8. The schematic diagram of led-milR-21 and the sequence of precursor gene PremilR21. A. led-milR-21 precursor sequence; B. prediction of led-milR-21 precursor structure; C. the genomic position of led-milR-21. Note: the sequence of led-milR-21 was shown in A in red color, in B in black circle and in C in red rectangle, respectively.

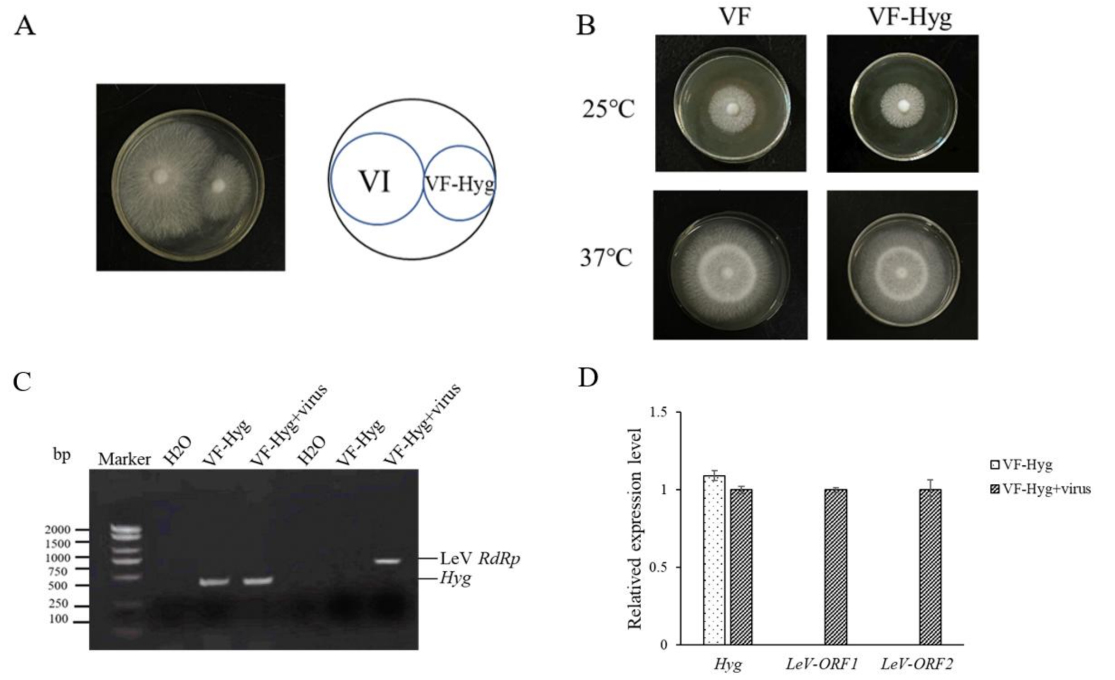

Fig.S9. Horizontal transmission of LeV from the VI strain to the the VF-Hyg strain via hyphal contact and verification. A. Horizontal transmission of LeV; B. The evaluation of the effect of introduction of *Hyg* on the cultural characteristics of VF-Hyg; C. RT-PCR verification of the reconstructived VF-Hyg and VF-Hyg+virus stains; D. RT-qPCR verification of the reconstructived VF-Hyg+virus stain.

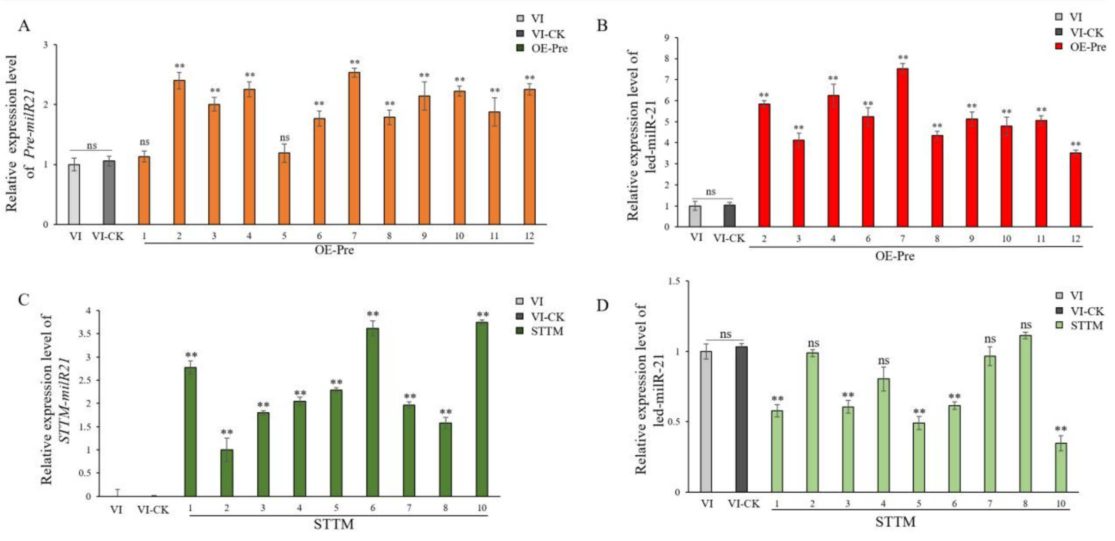

Fig.S10. RT-qPCR verification of the relative gene expression levels in obtained OEPre and SMMT transformants. A. Relative expression levels of Pre-milR21 in OEPre transformants; B. Relative expression levels of led-milR21 in OEPre transformants; C. Relative expression levels of *SMMT* in STTM transformants; Relative expression levels of led-milR21 in STTM transformants. Note: “ns” represents no significant differences, “**” represents extremly significant difference, $p < 0.01$.

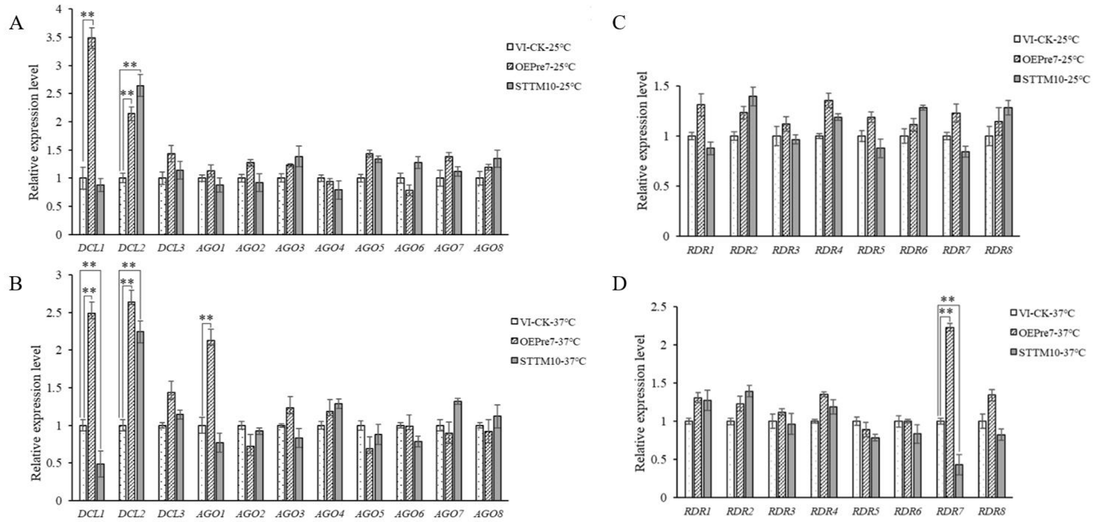

Fig.S11. RT-qPCR analysis of the expression of the RNAi key genes in the obtained OEPre and SMMT transformants under different temperature treatments. Note: "**" represents extremly significant difference, $p < 0.01$.

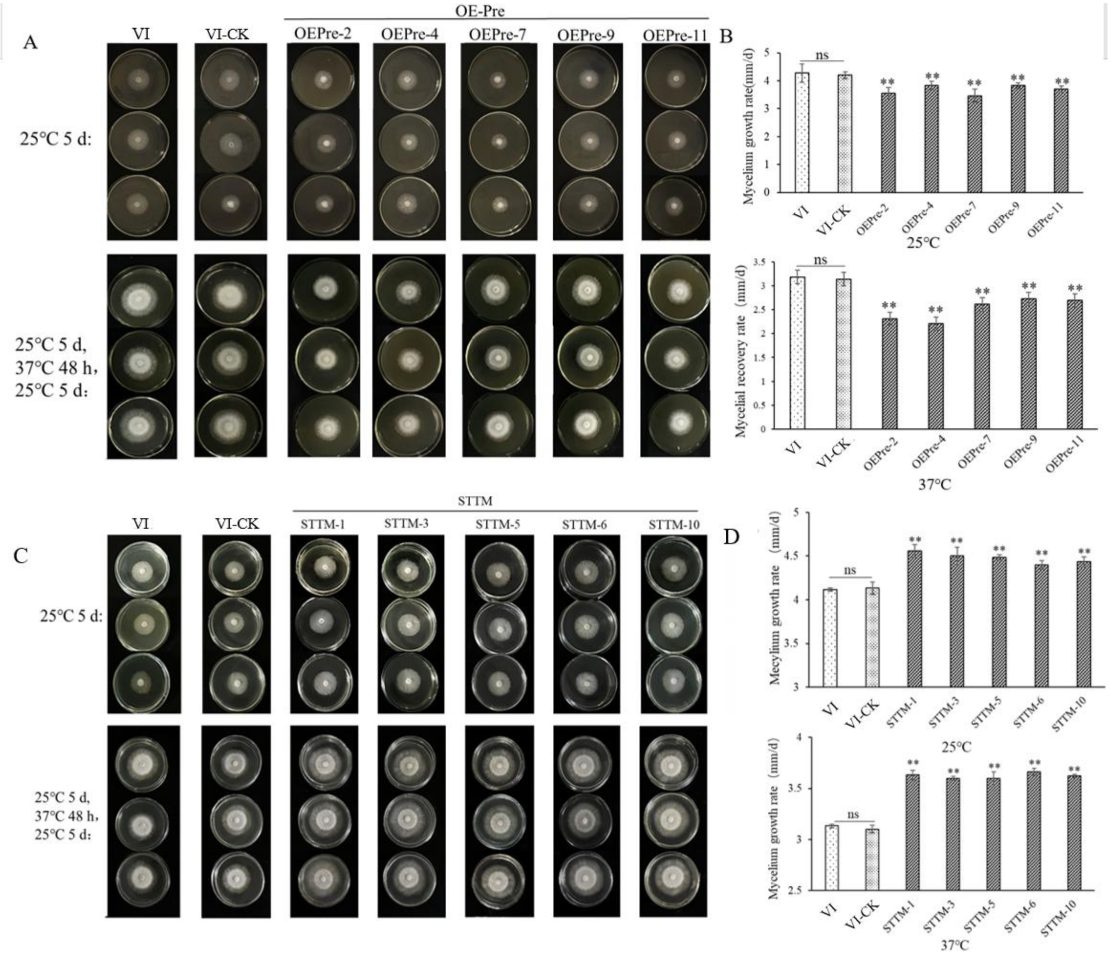

Fig. S12. The results of mycelial growth rate and thermotolerance of **the obtained OEPre and SMMT transformants under different temperature treatments.** A. The colonies phenotypic characteristics in the obtained OEPre and the control strains; B. The mycelial growth rate of the obtained OEPre and the control strains; C. The colonies phenotypic characteristics in the obtained SMMT and the control strains; D. the mycelial growth rate of the obtained SMMT and the control strains. Note: "ns" represents no significant differences, "**" represents extremly significant difference, $p < 0.01$.

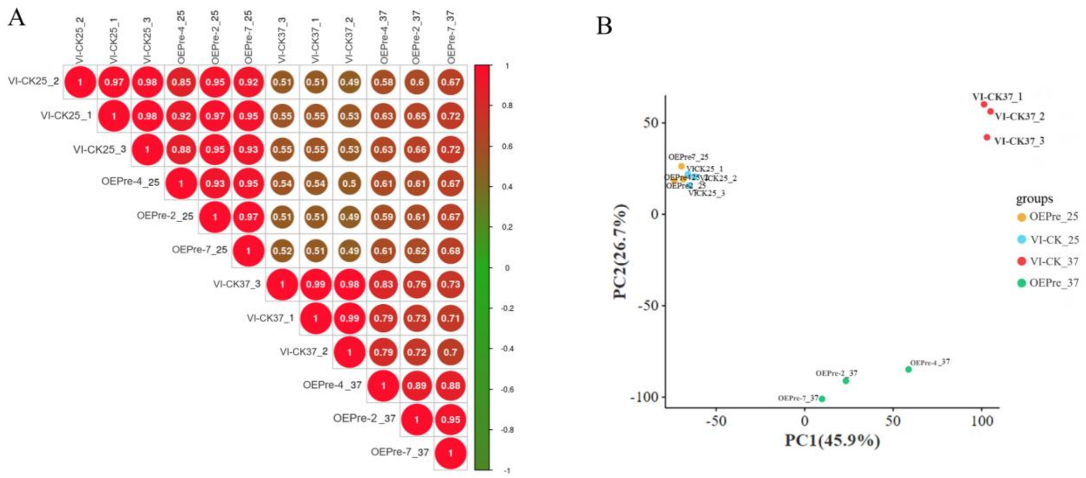

Fig.S13. Correlation coefficient and principal component analyses of the sequenced VI-CK and OEPre strains. A. The results of correlation coefficient analysis; B. The results of principal component analysis. Note: -1, -2, -3 mean three biological replicates.

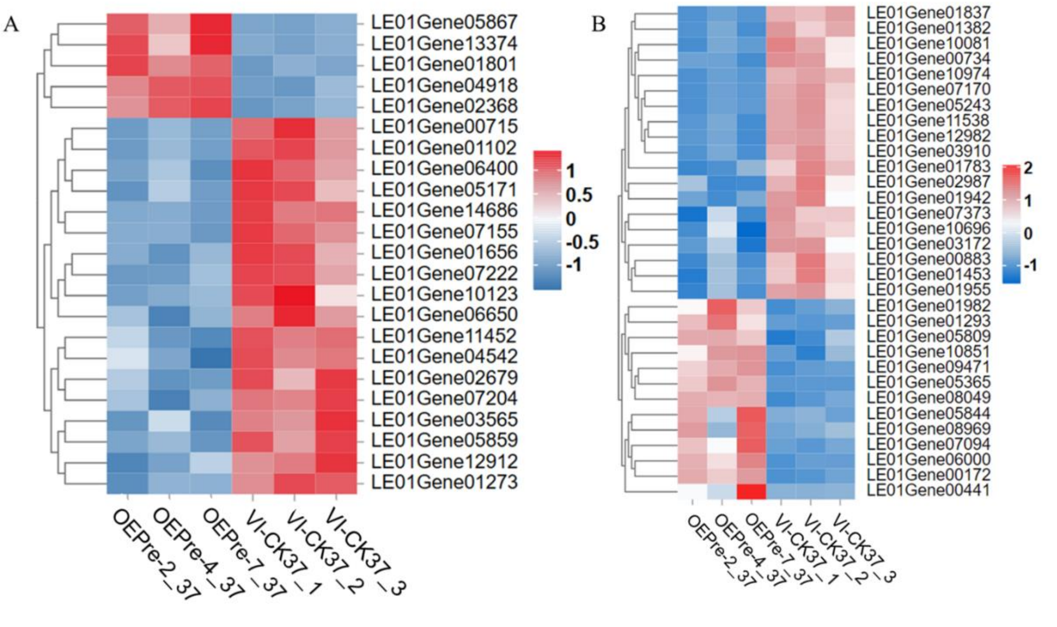

Fig.S14. The heat map of differential expressed heat shock response related genes (*HSPs*) and heat shock response related transcription factors (*HSFs*) in OEPre_37 vs VI-CK_37. A. *HSPs* ; B. *HSFs* .

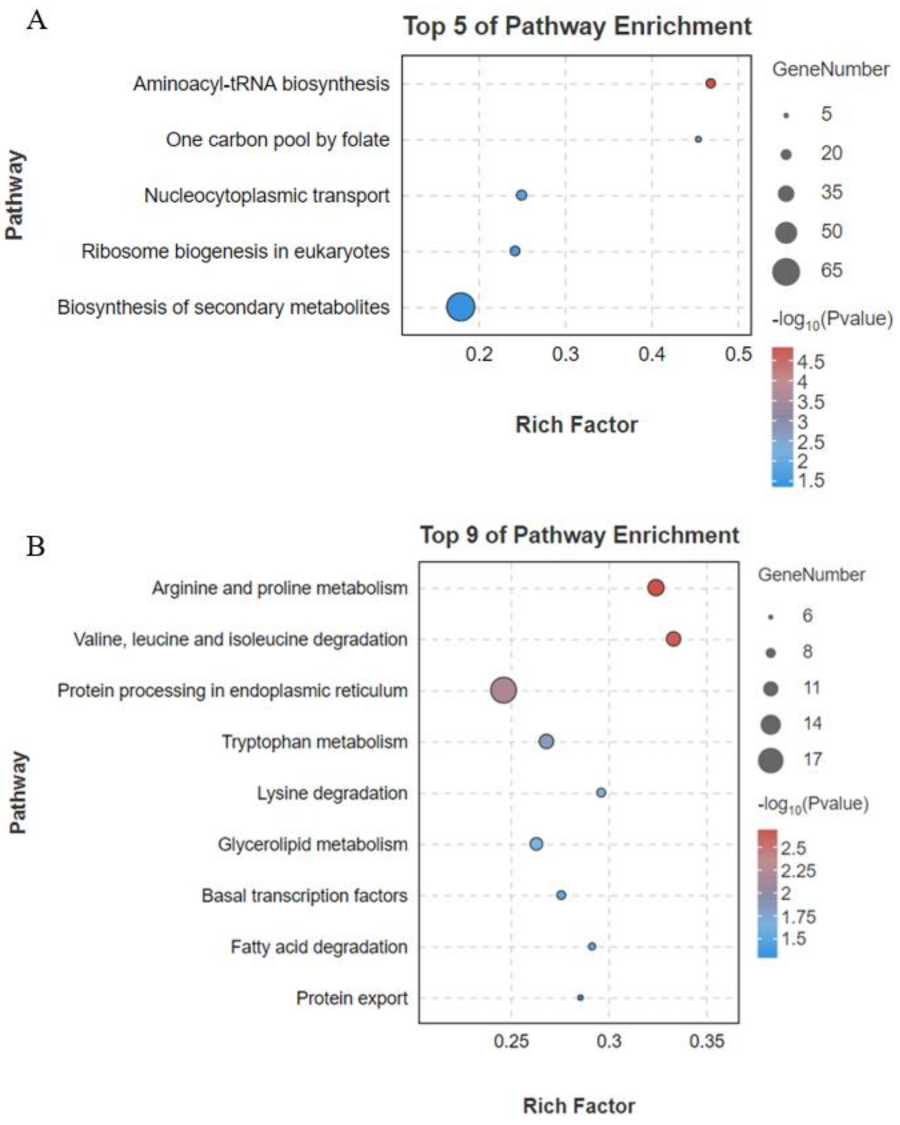

Fig.S15. The top KEGG enrichment pathways of DEGs in OEPre_37 vs VI-CK_37 after heat stress (HS). A. The top KEGG enrichment pathways of upregulated DEGs in OEPre_37 vs VI-CK_37 after HS; B. The top KEGG enrichment pathways of downregulated DEGs in OEPre_37 vs VI-CK_37 after HS.

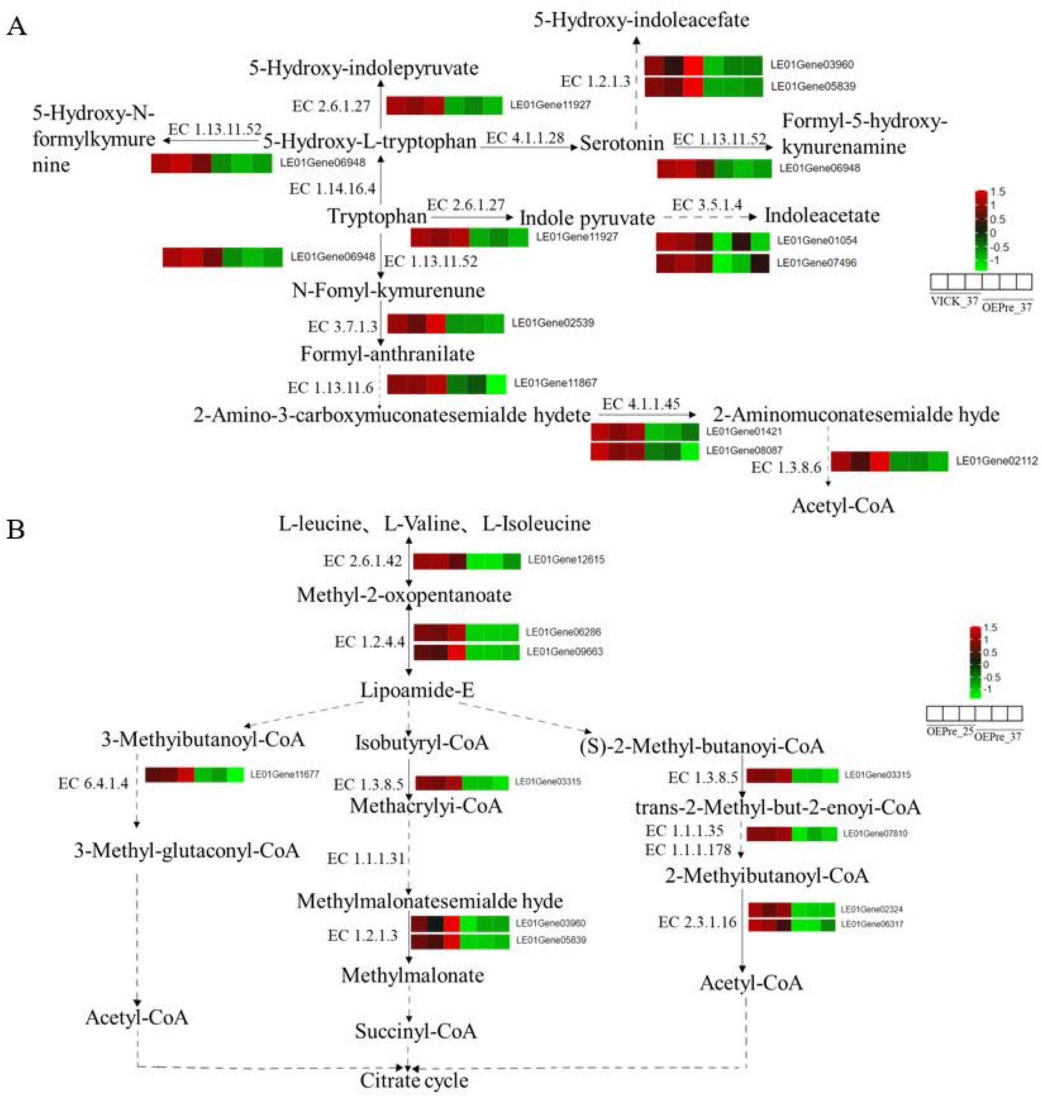

Fig.S16. The maps of top KEGG pathways in OEPre_37 vs VICK_37. A. Tryptophan metabolic pathway; B. Protein synthesis pathway of valine, leucine and isoleucine degradation.Note: Solid arrows indicate direct correlations between metabolites, dashed arrows indicate indirect correlations.

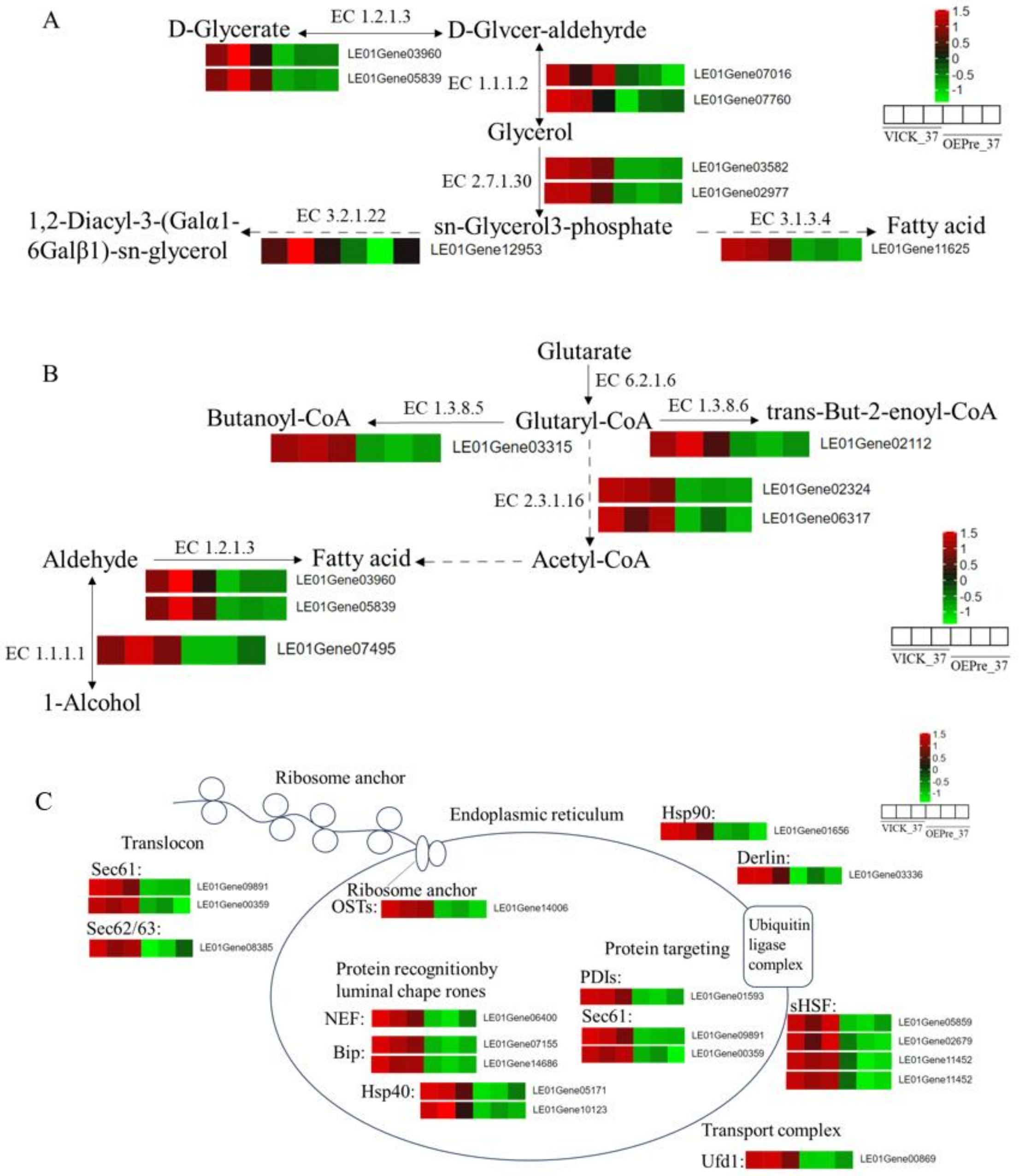

Fig. S17. The maps of top KEGG pathways in OEPre_37 vs VICK_37. A. Glycolipid Metabolism Pathway; B. Fatty Acid Degradation Pathway; C. Protein synthesis pathway of endoplasmic reticulum. Note: Solid arrows indicate direct correlations between metabolites, dashed arrows indicate indirect correlations.

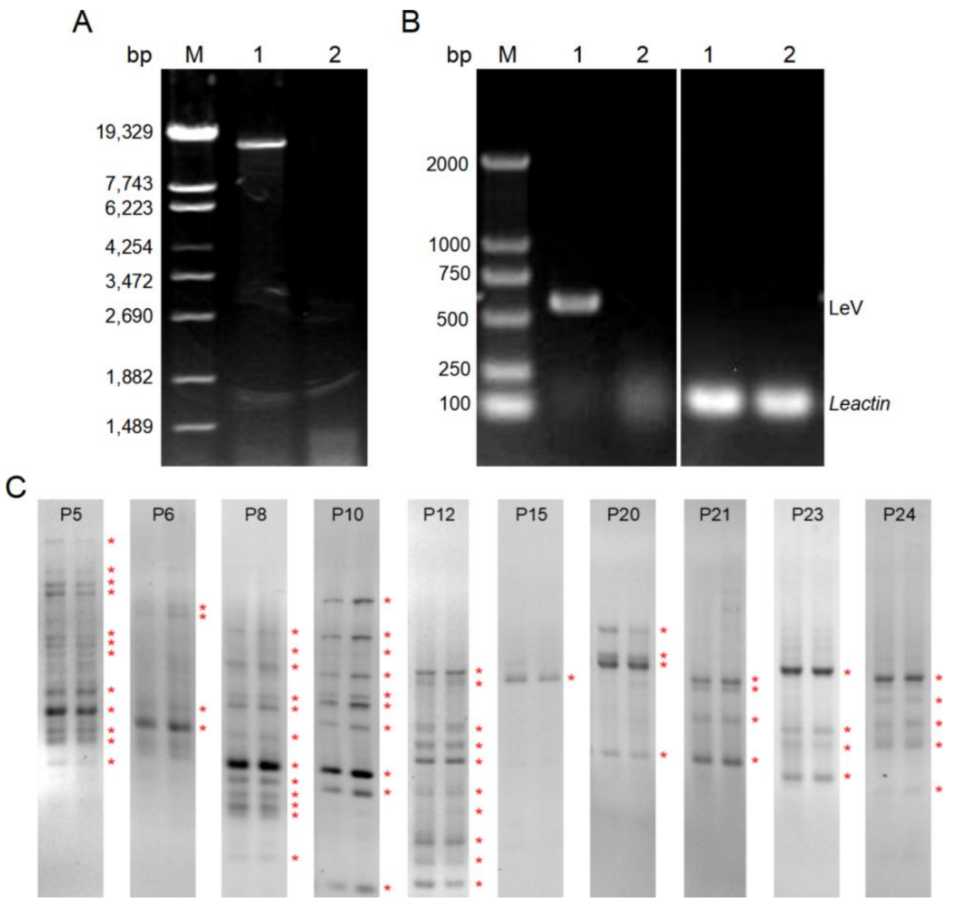

Fig.S18. The verified result of the elimination of LeV and the molecular fingerprints of the VI and VF strains. A. dsRNA verification; B. RT-PCR verification; C. ISSR analysis of VI and VF strain . Lane "1" and Lane "2" in (A) and (B) represent VI and VF, respectively.

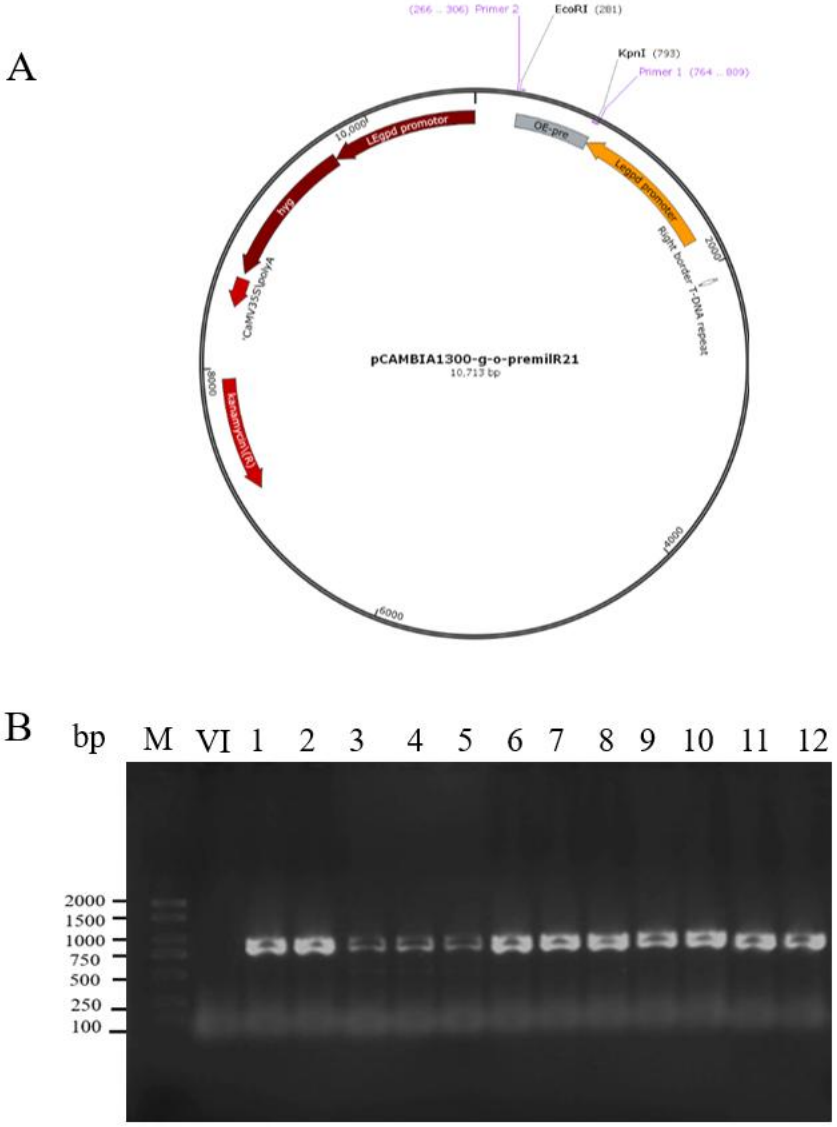

Fig.S19. A vector map and verification of recombinant plasmid pCAMBIA1300-g-o-*PremilR21*. A. A vector map of recombinant plasmid pCAMBIA1300-g-o-*PremilR21*; B. Electrophregrams of PCR products of putative OEPre transformants.

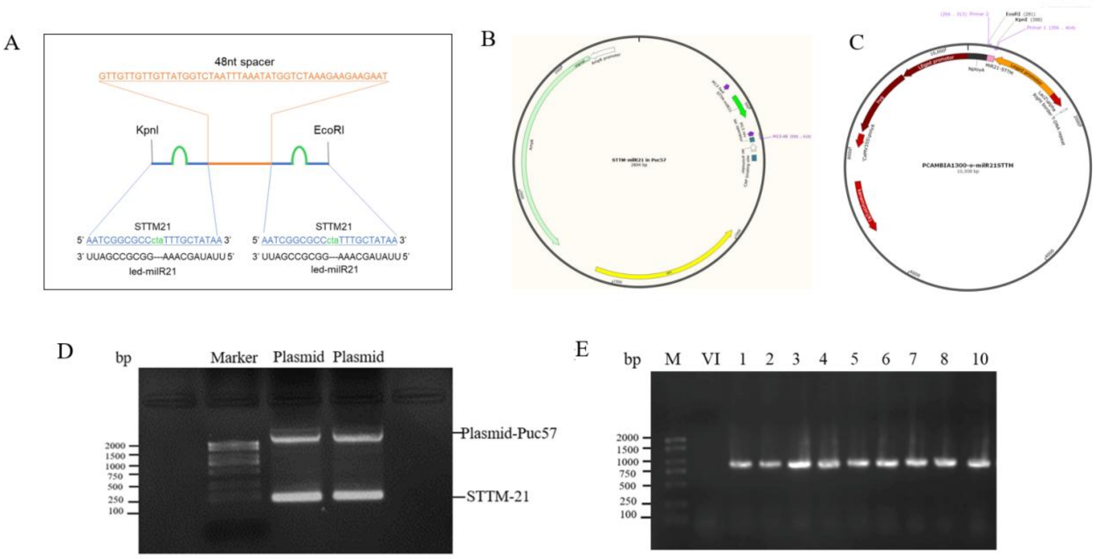

Fig.S20. Construction of recombinant plasmid PUC57-STTM and verification of STTM transformants. A. STTM-milR21 sequence. In two segments of led-milR-21 complementary sequences, three bases (CTA) were added to each, forming two partially complementary sequences. These were connected by a specific 48nt sequence, resulting in a designed STTM-milR-21 sequence with a total length of 94 bp; B. Recombinant plasmid PUC57-STTM. This STTM-milR21 sequence was synthesized by a company and inserted into the PUC57 plasmid; C. Recombinant plasmid pCAMBIA1300-o-STTM-milR-21.The plasmid pCAMBIA1300 with the Legpd promoter was used as the basic framework for the STTM expression vector, and the double restriction sites KpnI and EcoRI were used to insert the STTM-milR-21 gene fragment via homologous recombination; D. Enzyme digested of recombinant plasmid PUC57-STTM; E. The PCR analysis of putative transformants.

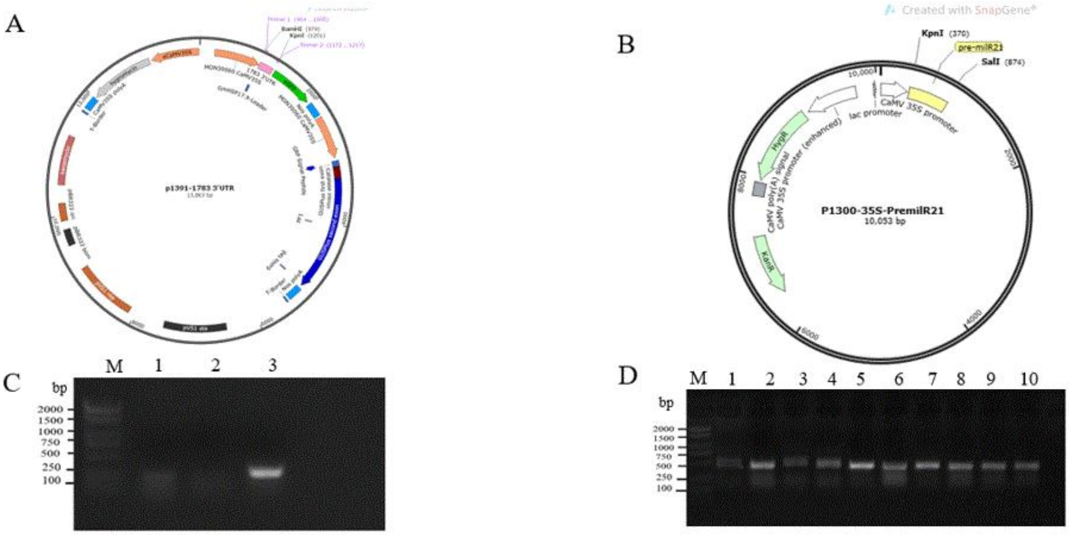

Fig.S21. Vector construction and verification of tobacco fluorescence transient expression system. A. The structure of vector P1391-1783-eGFP; B. The structure of constructed vector P1300-35S-premilR21; C. PCR verification of *Agrobacterium* transformants with P1391-1783-eGFP; D. PCR verification of *Agrobacterium* transformants with P1300-35S-premilR21.
